# Photolysis of Staphyloxanthin in Methicillin‐Resistant *Staphylococcus aureus* Potentiates Killing by Reactive Oxygen Species

**DOI:** 10.1002/advs.201900030

**Published:** 2019-03-30

**Authors:** Pu‐Ting Dong, Haroon Mohammad, Jie Hui, Leon G. Leanse, Junjie Li, Lijia Liang, Tianhong Dai, Mohamed N. Seleem, Ji‐Xin Cheng

**Affiliations:** ^1^ Department of Chemistry Boston University Boston MA 02215 USA; ^2^ Department of Comparative Pathobiology College of Veterinary Medicine Purdue University West Lafayette IN 47907 USA; ^3^ Prof. J.‐X. Cheng, Department of Electrical and Computer Engineering Boston University Boston MA 02215 USA; ^4^ Wellman Center for Photomedicine Massachusetts General Hospital Harvard Medical School MA 02114 USA; ^5^ State Key Laboratory of Supramolecular Structure and Materials Institute of Theoretical Chemistry Jilin University Changchun 130012 China; ^6^ Department of Biomedical Engineering Boston University Boston MA 02215 USA; ^7^ Photonics Center Boston University Boston MA 02215 USA

**Keywords:** methicillin‐resistant Staphylococcus aureus (MRSA), phototherapy, staphyloxanthin photobleaching, transient absorption microscopy

## Abstract

Confronted with the severe situation that the pace of resistance acquisition is faster than the clinical introduction of new antibiotics, health organizations are calling for effective approaches to combat methicillin‐resistant *Staphylococcus aureus* (MRSA) infections. Here, an approach to treat MRSA through photolysis of staphyloxanthin, an antioxidant residing in the microdomain of *S. aureus* membrane, is reported. This photochemistry process is uncovered through transient absorption imaging and quantitated by absorption spectroscopy, Raman spectroscopy, and mass spectrometry. Photolysis of staphyloxanthin transiently elevates the membrane permeability and renders MRSA highly susceptible to hydrogen peroxide attack. Consequently, staphyloxanthin photolysis by low‐level 460 nm light eradicates MRSA synergistically with hydrogen peroxide and other reactive oxygen species. The effectiveness of this synergistic therapy is well validated in MRSA planktonic culture, MRSA‐infected macrophage cells, stationary‐phase MRSA, persisters, *S. aureus* biofilms, and two mice wound infection models. Collectively, the work demonstrates that staphyloxanthin photolysis is a new therapeutic platform to treat MRSA infections.

## 1 Introduction

*Staphylococcus aureus* causes a variety of diseases ranging from skin and soft tissue infections to life‐threatening bacteremia.^1^ Moreover, *S. aureus* has acquired resistance to multiple antibiotic classes that were once effective.^2^ A classic example is the emergence of clinical isolates of methicillin‐resistant *S. aureus* (MRSA) strains in the 1960s that exhibited resistance to β‐lactam antibiotics.^3^ More recently, some MRSA strains have exhibited reduced susceptibility to newer antibiotics such as daptomycin and to last‐resort antibiotics such as vancomycin and linezolid.^4^ Besides the acquired resistance through mutational inactivation, *S. aureus* develops other strategies to undermine the effect of antibiotics, such as residing inside host immune cells,^5^ forming biofilms, and becoming dormant persisters.^6^ Those situations pose an appalling challenge to developing new ways to treat MRSA infections.

On the verge of post‐antibiotic area, researchers are taking several approaches to tackle MRSA‐caused infections. Repurposing existing anticancer, antifungal, and anti‐inflammatory drugs, has been pursued by harnessing their established feasibility and antibacterial properties.^7^ Therapeutic application of bacteriophages offers another promising alternative to combat staphylococcal infections.^8^ In addition, novel approaches are developed through targeting MRSA‐specific virulence factors. More than 90% of all *S. aureus* clinical isolates generate a golden pigment, staphyloxanthin (STX).^9^ STX condenses in the functional membrane microdomain of *S. aureus*,^10^ endowing *S. aureus* with membrane integrity and excellent antioxidant property.^11^ Ever since Nizet and co‐workers elucidated the pivotal role of STX, the virulence factor which protects *S. aureus* from neutrophil‐based killing,^12^ stripping this important pigment off MRSA has become a novel therapeutic approach.^13^ A range of synthetic cholesterol inhibitors have been harnessed to inhibit STX biosynthesis.[[qv: 12,13b]] Chen et al. found that naftifine, an FDA‐approved antifungal drug, blocked the biosynthesis of STX through inhibition of diapophytoene desaturase.^14^ Jabra‐Rizk et al. demonstrated that sesquiterpene farnesol, a natural plant metabolite, effectively suppressed the production of STX through binding the active domain of the dehydrosqualene synthase, thus compromising the membrane integrity.^15^ However, administration of exogenous agents only achieved limited efficacy possibly due to off‐targeting.^16^ Therefore, drug‐free approaches to eradicate STX have been pressingly desired.

Here, through label‐free transient absorption imaging of nonfluorescent chromophores in *S. aureus*, we accidentally find that STX is prone to photolysis and this photolysis process strongly depends on the excitation wavelength. By absorption spectroscopy, we identify that the optimal wavelength for STX photolysis is around 460 nm. We also unveil that 460 nm light induces STX C=C bond breakdown by employing Raman spectroscopy and mass spectrometry. We then find that STX photolysis transiently elevates the membrane permeability and renders MRSA highly susceptible to reactive oxygen species attack. Based on these findings, we developed a highly effective synergy between STX photolysis and low‐concentration hydrogen peroxide, which is well established in eliminating stationary‐phase MRSA, MRSA persisters, *S. aureus* biofilms, and MRSA‐infected mice wound models. We also find that STX photolysis could assist macrophage cells to eliminate intracellular MRSA, whereas high‐concentration antibiotic fails. Our findings open new opportunities for treating MRSA infections.

## 2 Results and Discussion

### 2.1 STX Photobleaching Revealed under a Transient Absorption Microscope

**Figure 1:**
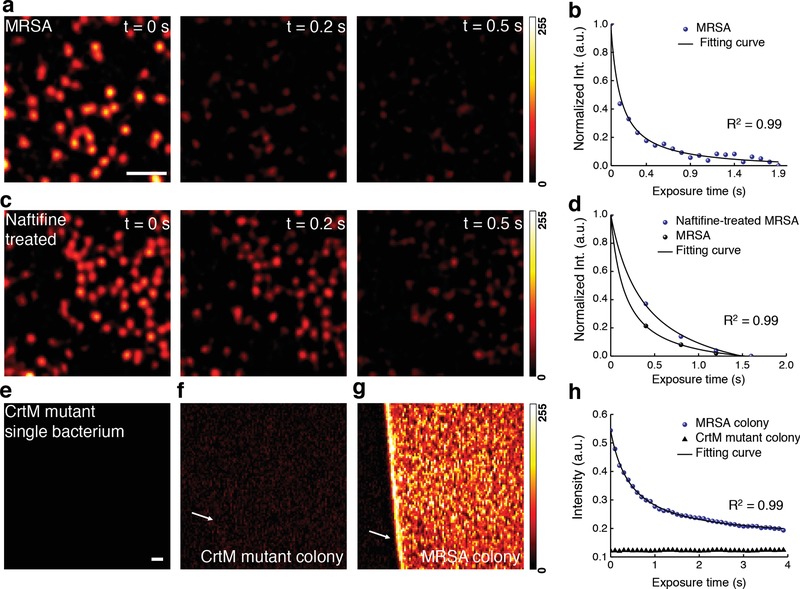
Photobleaching of staphyloxanthin in MRSA uncovered by transient absorption microscopy. a) Pseudocolor time‐lapse images of MRSA. Scale bar = 5 µm, applies to images in (a) and (c). b) Representative time‐lapse signal (normalized) from MRSA. c) Pseudocolor time‐lapse images of naftifine‐treated MRSA. d) Representative time‐lapse signals (normalized) from MRSA and naftifine‐treated MRSA. e–g) Pseudocolor images of CrtM mutant, CrtM mutant colony, and MRSA colony at *t* = 0 s, respectively. Scale bar = 20 μm, applies to (e–g). h) Representative raw time‐lapse signals from MRSA colony and CrtM mutant colony. White arrows indicate the interface between air and sample. Time‐lapse signals were fitted by Equation (1). Images were processed from raw data with dynamic range of 0–255 through ImageJ.

Initially we attempted to differentiate MRSA from methicillin‐susceptible *S. aureus* by transient absorption imaging (Figure S1, Supporting Information) of their intrinsic chromophores. Intriguingly, once the cultured MRSA was placed under the microscope, the strong signal measured at zero delay between the 520 nm pump and 780 nm probe pulses quickly attenuated over second scale (**Figure** **1**a; Movie S1, Supporting Information). We hypothesized that a specific chromophore in MRSA is prone to photobleaching under the abovementioned settings. To verify this, we fitted the time‐course curve with a photobleaching model developed for photosensitizers^17^ (Figure 1b, see Methods in the Supporting Information )

(1) $$y\;=\;y_{0}\;+\;A*\frac{\exp\left(-\frac{t}{\tau_{1}}\right)}{1\;+\;\frac{\tau_{1}}{\tau_{2}}*\left(1-\exp\left(-\frac{t}{\tau_{1}}\right)\right)}$$

where *t* is the duration of light irradiation, *y* is the signal intensity, *y*_0_ and *A* are constants, τ_1_ and τ_2_ are the time constants for the first‐ and second‐order photobleaching, respectively. The first‐order bleaching occurs at low concentration of chromophores (τ_2_ =  ∞). The second‐order bleaching takes place when quenching within high‐concentration surrounding chromophores dominates (τ_1_ =  ∞, Figure S2, Supporting Information). Derivation of Equation (1) is detailed in the Supporting Information. Strikingly, this photobleaching model fitted well with the raw time‐course curve (τ_1_ =  ∞, τ_2_ = 0.15 ± 0.02 s). Moreover, oxygen depletion (Na_2_S_2_O_4_: oxygen scavenger) showed negligible effect on the photobleaching speed (τ_2_ = 0.14 ± 0.01 s, Figure S3a, Supporting Information). The same phenomenon was observed in methicillin‐susceptible *S. aureus* (Figure S3b, Supporting Information). To determine whether oxygen plays an essential role during this photobleaching process, we kept the extracted chromophore solution bubbling with nitrogen gas for 2 h in order to deplete the oxygen.^18^ We found that oxygen depletion did not affect the photobleaching process (Figure S3c, Supporting Information). Collectively, these data support a second‐order photobleaching process.

Next, we aimed to deduce the specific chromophore inside MRSA that accounts for the observed photobleaching phenomenon. It is known that carotenoids are photosensitive due to the conjugated C=C bonds.^19^ Therefore, we hypothesized that STX, the major carotenoid pigment residing in the cell membrane of MRSA, underwent photobleaching in our transient absorption study. To test this hypothesis, we treated MRSA with naftifine to block the synthesis of STX.^14^ The treated MRSA exhibited lower signal intensity (Figure 1c) and slower photobleaching speed (Figure 1d). Specifically, τ_2_ of naftifine‐treated MRSA (0.39 ± 0.07 s) is 2.5 times of that of MRSA (0.15 ± 0.02 s), in consistence with second‐order photobleaching. Furthermore, no transient absorption signal was observed in *S. aureus* strain with mutation in dehydrosqualene synthase (CrtM) (Figure 1e) that is responsible for STX biosynthesis.^11^ To avoid the systematic error aroused by single bacterium measurement, we repeated the same analysis using bacterial colonies. It turned out that CrtM mutant colony (Figure 1f,h) only exhibited background induced by cross‐phase modulation,^20^ whereas the MRSA colony showed a sharp contrast against the background (Figure 1g) and a fast photobleaching decay (Figure 1h). Taken together, these data confirm that STX in MRSA accounts for the observed photobleaching phenomenon.

### 2.2 Wide‐Field Photobleaching of STX by a Portable Device

In the transient absorption study, when changing 520 nm pump irradiance while fixing 780 nm probe intensity, both signal intensity and τ_2_ changed drastically (Figure S4a,c, Supporting Information), whereas the alteration of probe irradiance only affected the transient absorption signal intensity but not τ_2_ (Figure S4b,d, Supporting Information). These findings collectively imply that photobleaching efficacy is highly dependent on the excitation wavelength (Figure S4e, Supporting Information), which is consistent with the fact that photobleaching is grounded on the absorption of chromophore.^21^

**Figure 2:**
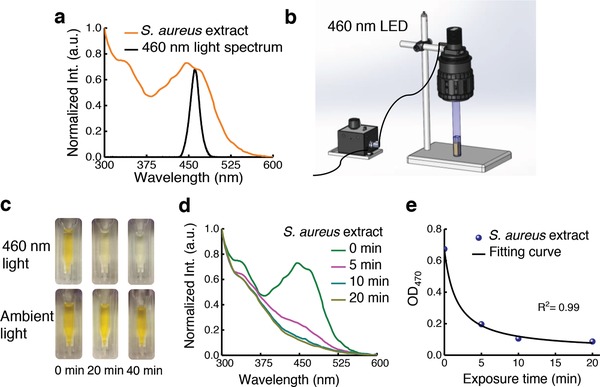
Staphyloxanthin is prone to photobleaching under blue light irradiance. a) Absorption spectrum of crude STX extract (brown) and emission profile of a blue LED (black). b) Schematic illustration of a portable LED‐based wide‐field photobleaching device. c) Pictures of crude STX extract exposed to 460 nm light and ambient light at different time intervals. d) Absorption spectra of crude STX extract over different 460 nm light exposure time. e) OD of crude STX extract at 470 nm adapted from (d) over 460 nm light exposure time. Data points were fitted by Equation (1).

To find the optimal wavelength for bleaching STX, we measured the absorption spectrum of crude STX extract from *S. aureus*. The extract showed strong absorption in the window from 400 to 500 nm (**Figure** **2**a). Based on this result, we built a portable device composed of a blue LED with a central emission wavelength at 460 nm for wide‐field bleaching STX (Figure 2b). We exposed the crude STX extract to the 460 nm light (intensity, 90 mW cm^−2^) for different time intervals. Remarkably, the distinctive golden color of STX disappeared within 30 min exposure, whereas the control group under ambient light remained unchanged (Figure 2c). Its absorption within 400–500 nm window decreased dramatically over 460 nm light exposure (Figure 2d). The optical density at 470 nm (from Figure 2d) versus the 460 nm light dose can be well fitted with Equation (1) (Figure 2e). Additionally, naftifine‐treated or CrtM‐mutant MRSA extracts were insensitive to 460 nm light exposure, indicated by their nearly unchanged absorption spectra (Figure S5a–c, Supporting Information). These findings collectively suggest that STX is prone to photobleaching under 460 nm light irradiance.

### 2.3 Mass Spectrometry and Raman Spectroscopy Unveil the Photochemistry of STX under 460 nm Light Irradiance

**Figure 3:**
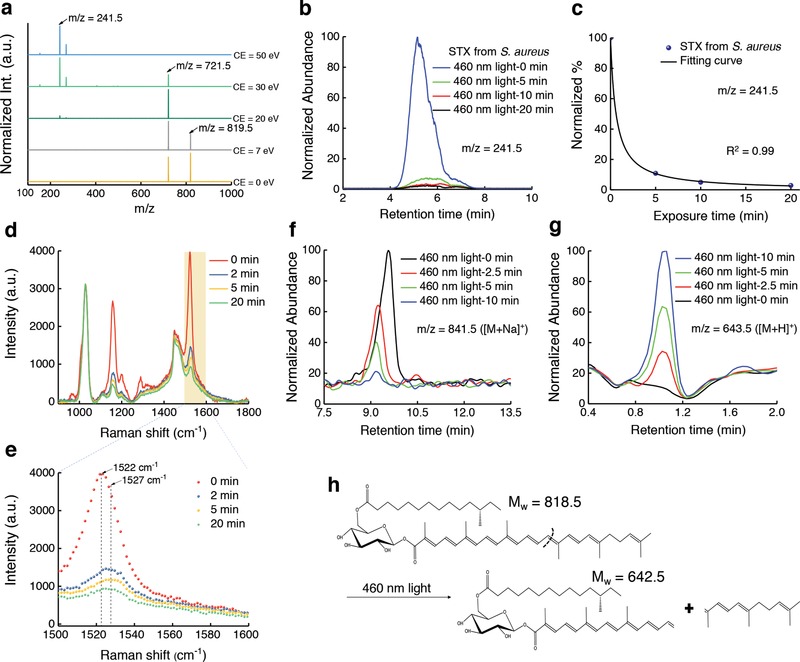
Mass spectrometry and Raman spectroscopy unveil the photochemistry of staphyloxanthin under 460 nm light exposure. a) MS spectra of crude STX extract at different collision energy (CE). Peaks of *m/z* = 819.5, 721.5, and 241.5 are highlighted by black arrows. b) HPLC chromatograms of STX extracted from concentrated MRSA over 460 nm light exposure at an intensity of 90 mW cm^−2^. c) The amount of STX calculated from (b). Quantification of STX is determined from the peak area of STX in HPLC chromatograms shown in (b). Data points are fitted by Equation (1). d) Raman spectra of crude STX (extracted from concentrated MRSA) under different 460 nm light doses. 460 nm light intensity: 200 mW cm^−2^. Raman excitation wavelength: 532 nm, acquisition time: 30 s. e) Zoomed‐in view of (d) in the Raman shift range from 1500 to 1600 cm^−1^, Raman shifts at 1522 and 1527 cm^−1^ are highlighted by black arrows and dashed lines. f,g) UPLC chromatograms of STX f) and one of its corresponding products g) over 460 nm light exposure. h) Tentative breakdown pathway of STX under 460 nm light irradiance.

To understand the chemical nature of this photobleaching process, we investigated the STX degradation products induced by 460 nm light irradiation via mass spectrometry (MS). Figure S6 in the Supporting Information presents the MS spectrum of MRSA extract with *m/z* ranging from 200 to 1000 eV at a collision energy of 10 eV. An abundant peak appears at *m/z* = 721.5, while a weaker peak at *m/z* = 819.5 ([M+H]^+^) is consistent with the molecular weight of STX (*M*_w_ = 818.5 g mol^−1^). To find out the relationship between *m/z* = 721.5 and 819.5, we gradually increased the collision energy from 0 to 20 eV. In **Figure** **3**a, the abundance of *m/z* = 721.5 increases relative to that of *m/z* = 819.5 with increasing collision energy, indicating that *m/z* = 721.5 is a product ion from *m/z* = 819.5. When the collision energy was higher than 30 eV, *m/z* = 241.5, a product of the precursor ion *m/z* = 721.5, became dominant and presented as a stable marker (Figure 3a). Thus, to accurately quantify the amount of STX versus 460 nm light dose, we targeted the peak area in high‐performance liquid chromatography (HPLC) chromatograms specifically from ion *m/z* = 241.5 (Figure 3b). Figure 3c depicts the bleaching dynamics of STX induced by 460 nm exposure. 5 min 460 nm light exposure (dose: 27 J cm^−2^) decomposed 90% of STX extracted from ≈10^9^ colony‐forming units (CFU mL^−1^) MRSA, and a dose of 54 J cm^−2^ bleached all the STX pigments (data not shown). In contrast, extracts from naftifine‐treated and CrtM‐mutant *S. aureus* had negligible response to 460 nm light exposure as shown in Figure S5d–f in the Supporting Information.

Next, we employed Raman spectroscopy to elucidate how 460 nm light irradiance degrades STX. STX exhibited an abundant peak at the Raman shift of 1522 cm^−1^ (Figure 3d), which was assigned to bacterial carotenoids.^22^ As the duration of 460 nm light exposure increases, the Raman intensity at 1522 cm^−1^ dramatically decreases (Figure 3d). Similar phenomenon was observed in single MRSA (Figure S7, Supporting Information). Notably, we found an ≈5 cm^−1^ increase in Raman shift after 460 nm light exposure (Figure 3e), which likely results from a decreased number of conjugated C=C bonds^23^ during the photobleaching process. It was worth noting that the protein content (indicated by Raman shift at around 1000 cm^−1^) remained unchanged during the photobleaching process (Figure 3e). These findings suggest that 460 nm light irradiance breaks down the STX molecule.

We further utilized time‐of‐flight MS/MS to quantitate this photolysis process. Different from the *m/z* = 819.5 peak where STX locates in the HPLC chromatograph, STX displays a main peak at *m/z* = 841.5 in the ultraperformance liquid chromatography (UPLC) chromatograms (Figure 3f), which is an adjunct between STX and Na^+^ ([M+Na]^+^). Degradation of STX would bolster the aggregation of chemical segments. Accordingly, we screened a patch of products after STX degradation (Figure S8, Supporting Information). In particular, the intensity of the peak at *m/z* = 643.5 corresponding to an adjunct between an STX segment with H^+^ ([M+H]^+^) significantly increased as 460 nm light exposure elongated (Figure 3g). Figure 3h suggests a potential mechanism of how this segment could be formed from breakdown of the C=C bond in STX after 460 nm light exposure. These findings underline that STX can be photolyzed by 460 nm light. We note that the interpretation of other products (Figure S8, Supporting Information) necessitates further in‐depth analysis.

### 2.4 STX Photolysis Alone Is Not Sufficient to Eradicate MRSA

**Figure 4:**
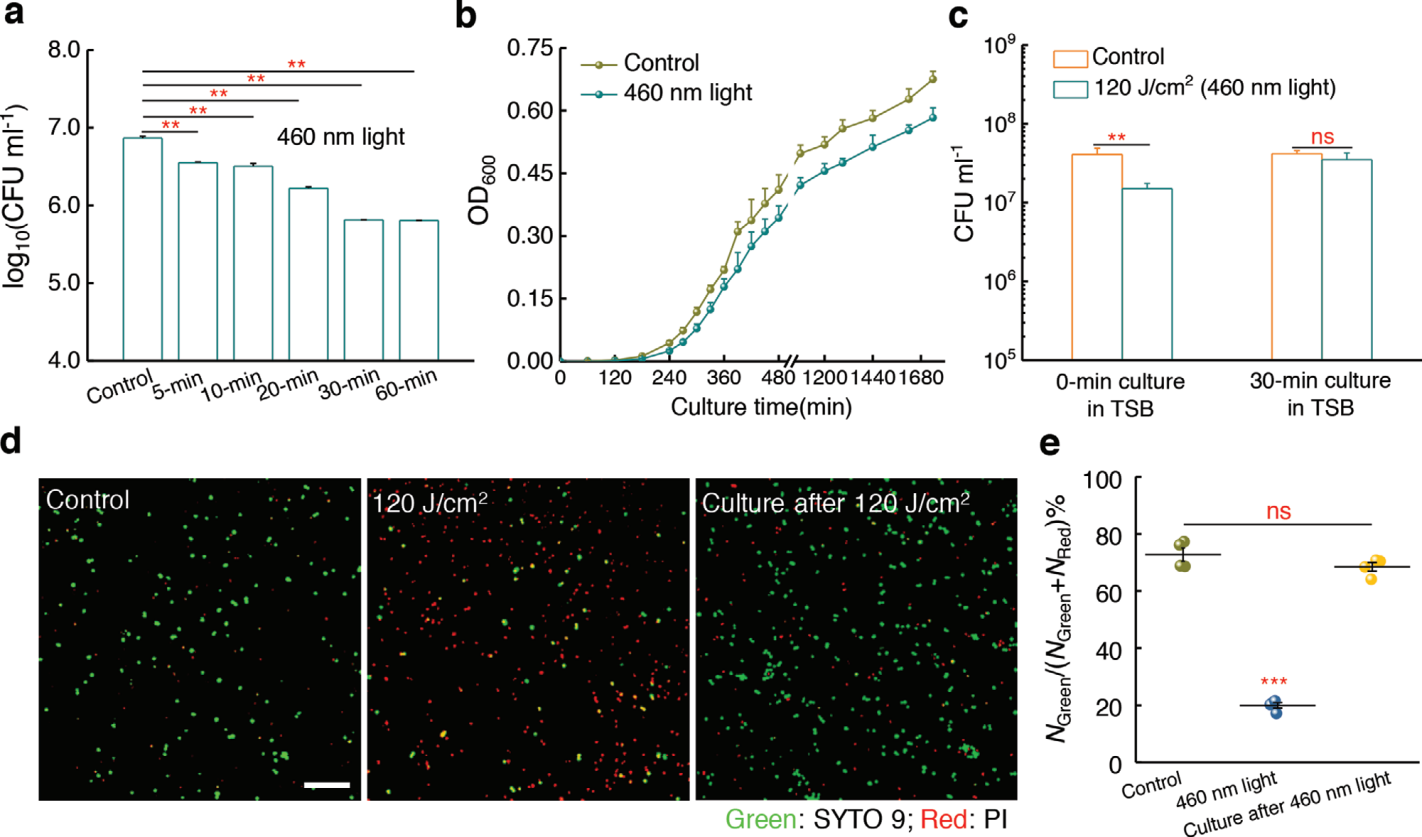
Staphyloxanthin photolysis transiently elevates MRSA membrane permeability and is unable to eradicate MRSA completely. a) Log change in MRSA colony‐forming units (CFU mL^−1^) after treatment with 460 nm light at different doses. Intensity: 60 mW cm^−2^, *N* = 3. b) Growth curves of MRSA after no treatment (control) or treatment with 120 J cm^−2^ 460 nm light irradiance (*N* = 6). c) MRSA CFU mL^−1^ after no treatment (control) or treatment with 120 J cm^−2^ 460 nm light irradiance, and 30 min culture in TSB medium after 120 J cm^−2^ 460 nm light irradiance (*N* = 3). d) Confocal laser scanning imaging of membrane permeability after no treatment (control) or treatment with 120 J cm^−2^ 460 nm light, and 30 min culture in TSB medium after 120 J cm^−2^ 460 nm light irradiance. Scale bar = 10 µm. Green: intact membrane; Red: damaged membrane. e) Statistical analysis of cell membrane permeability for (d). *N*_Green_ and *N*_Red_ are the number of MRSA with intact membrane and damaged membrane, respectively (*N* = 4). Error bars show standard error of the mean (SEM). Unpaired two‐tailed *t*‐test (***: *p* < 0.001, **: *p* < 0.01, ns: not significant).

Given that STX is critical to the integrity of *S. aureus* cell membrane,^11^ we wondered whether photolysis of STX could eliminate MRSA. Blue light at 405 and 470 nm have been used to kill MRSA, as reviewed by Wang et al.^24^ However, the efficacy is limited and the molecular mechanism remains elusive. We have established above that STX is the molecular target of blue light irradiation. Accordingly, we found that increasing 460 nm light dose steadily decreased the level of MRSA CFU mL^−1^ (**Figure** **4**a). Moreover, MRSA was more sensitive to 460 nm light exposure than the CrtM mutant (Figure S9, Supporting Information). Nevertheless, the killing efficiency saturates at a level of 216 J cm^−2^ (Figure 4a). To investigate the reason, we continuously monitored the growth of MRSA in fresh medium after 10 min 460 nm light exposure. Remarkably, MRSA exposed to 460 nm light was able to recover and multiply after being cultured in medium (Figure 4b). We also measured CFU mL^−1^ of 460 nm light‐exposed MRSA after being cultured in fresh medium for 30 min. It seems that 460 nm light puts MRSA in a “traumatized” state other than a death form, and the traumatized MRSA could recover and multiply quickly in the fresh medium (Figure 4c). Since STX is enriched in membrane microdomain and is essential for membrane integrity,^11^ we conducted a membrane permeability assay^25^ before and after STX photolysis. We found that the membrane permeability from 460 nm light‐exposed MRSA significantly increased compared to control group (Figure 4d,e). However, light‐exposed MRSA is able to recover the integrity of cell membrane after being cultured in fresh medium within 30 min (Figure 4d,e). Together, these observations suggest that STX photolysis alone is not sufficient to kill MRSA completely.

### 2.5 STX Photolysis and Hydrogen Peroxide Attack Synergistically Eradicate Planktonic MRSA

**Figure 5:**
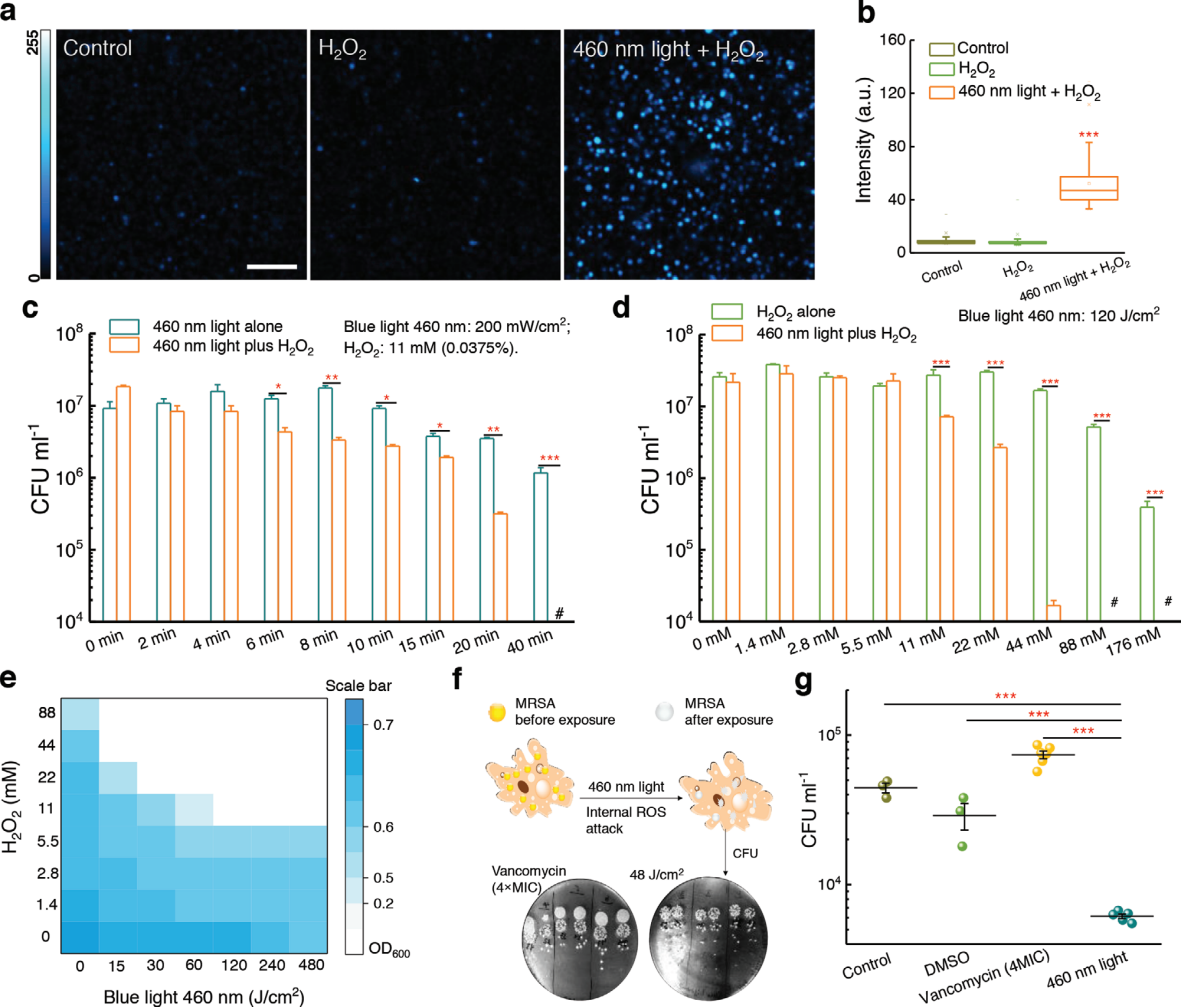
Staphyloxanthin photolysis and reactive oxygen species synergistically eliminate planktonic MRSA and intracellular MRSA. a) Fluorescence images from confocal laser scanning imaging of intracellular H_2_O_2_ after no treatment (control), 0.15% H_2_O_2_, or 460 nm light combined with 0.15% H_2_O_2_. Scale bar = 10 µm. b) Box chart analysis of fluorescence intensity in (a). c) MRSA CFU mL^−1^ after treatment with 460 nm light alone, and treatment with H_2_O_2_ plus 460 nm light (*N* = 3). d) MRSA CFU mL^−1^ after treatment with H_2_O_2_ alone, and treatment with 460 nm light plus H_2_O_2_ (*N* = 3). e) Checkerboard broth microdilution assays showing the dose‐dependent potentiation of H_2_O_2_ by 460 nm light irradiation against MRSA USA300. f) Schematic illustrating the utilization of 460 nm light to treat intracellular MRSA. Pictures of spread plates from vancomycin (4× MIC) and 460 nm light‐treated groups are shown. g) CFU mL^−1^ results of intracellular MRSA after no treatment or treatment with vancomycin (4 × MIC), and 460 nm light (48 J cm^−2^) (*N* = 3–6). Error bars show SEM. Unpaired two‐tailed *t*‐test (***: *p* < 0.001, **: *p* < 0.01, ns: not significant).

We then asked whether STX photolysis could transiently enhance cellular uptake of hydrogen peroxide, one of the most common reactive oxygen species.^26^ We performed confocal laser scanning fluorescence imaging of *S. aureus* with a fluorescent probe (see Methods in the Supporting Information) to image intracellular hydrogen peroxide. It turned out that after 460 nm light exposure, H_2_O_2_‐treated MRSA exhibited a much stronger fluorescence intensity than H_2_O_2_‐alone treated MRSA or untreated MRSA (**Figure** **5**a,b). No significant difference was found between H_2_O_2_‐alone treated MRSA and untreated MRSA (Figure 5b), indicating untreated MRSA has the capability to neutralize H_2_O_2_. This evidence proves that STX photolysis enhances the entry of hydrogen peroxide into the bacteria, which may cause intensified toxicity to MRSA.

To examine the bactericidal effect of STX photolysis when combined with hydrogen peroxide, we measured the viability of MRSA exposed to H_2_O_2_ after 460 nm light exposure. When MRSA was treated with 460 nm light (dose: 108 J cm^−2^) followed by increasing concentrations of H_2_O_2_, a significant reduction (*p* < 0.001) in CFU mL^−1^ was obtained (Figure 5c). Strikingly, 480 J cm^−2^ 460 nm light exposure combined with 0.0375% of H_2_O_2_ (culture time: 30 min) eradicated 10^7^ MRSA CFU mL^−1^ completely (Figure 5c). Therefore, we hypothesized that 460 nm light and H_2_O_2_ work synergistically to eradicate MRSA. To verify this synergistic effect, we performed the same measurements at various 460 nm light doses while fixing the concentration of H_2_O_2_ (Figure 5d). Then we calculated a fractional bactericidal concentration index (FBCI),^27^ which is widely used in the pharmaceutical research, to evaluate the combinational behavior between two agents. FBCI was calculated by using FBC that stands for fractional bactericidal concentration and MBC which defines the minimum bactericidal concentration. FBC of drug A = MBC of drug A in combination with drug B divided by MBC of drug A alone, FBC of drug B = MBC of drug B in combination with drug A divided by MBC of drug B alone. The FBCI = FBC of drug A + FBC of drug B. An FBCI of ≤0.5 is considered to demonstrate synergy, while an FBCI of 1.0 defines an additive effect. An FBCI > 2 defines antagonism. As shown in Figure 5c,d, 44 × 10^−3^ m of H_2_O_2_ along with 120 J cm^−2^ (20 min) eliminates around 99.9% the MRSA USA300. Since 200 mW cm^−2^ 460 nm light did not eradicate all the MRSA USA300 after 40 min exposure time (480 J cm^−2^), we have

(2) $$\mathrm{FBC}\;\mathrm{of}\;\mathrm{drug}\;A\;\left(\mathrm{blue}\;\mathrm{light}\right)\;<\;\frac{120\;J\;{\mathrm{cm}}^{-2}}{480\;J\;{\mathrm{cm}}^{-2}\;}\;=\;0.25$$

Since we found that 176 × 10^−3^ m H_2_O_2_ is not able to eliminate 99.9% of the bacteria, we have

(3) $$\mathrm{FBC}\;\;\mathrm{of}\;\mathrm{drug}\;\;B\left(H_{2}O_{2}\right)\;\;<\;\;\frac{44\;\times \;10^{-3}\;M\;}{176\;\times \;10^{-3}\;M\;}\;\;=\;\;0.25$$

Therefore, FBCI = FBC of blue light + FBC of H_2_O_2_ < 0.25 + 0.25 = 0.5. This result indicates that STX photobleaching works synergistically with hydrogen peroxide to eliminate MRSA USA300.

Next, we conducted a checkerboard broth microdilution assay^28^ to calculate the fractional inhibitory concentration index (FICI), which is another commonly used index to evaluate the combinational behavior. In this assay, FICI is calculated by using FIC which stands for fractional inhibitory concentration and MIC which defines minimal inhibitory concentration. FIC of drug A = MIC of drug A in combination with drug B divided by MIC of drug A alone, FIC of drug B = MIC of drug B in combination with drug A divided by MIC of drug B alone, and FICI index = FIC of drug A + FIC of drug B. An FICI of ≤0.5 is considered to demonstrate synergy. An FICI of 1.0 is defined as additive. Antagonism is defined as an FICI > 2.0. From Figure 5e, we can calculate the range of FICI

(4) $$\mathrm{FICI}\;<\;\frac{30\;J\;{\mathrm{cm}}^{-2}}{480\;J\;{\mathrm{cm}}^{-2}}\;+\;\frac{22\;\times \;10^{-3}\;M}{88\;\times \;10^{-3}\;M}\;=\;0.31$$

This data further confirms the synergy between STX photobleaching and H_2_O_2_ in treating MRSA USA300. Noteworthily, this treatment did not affect other species of staphylococci, such as *Staphylococcus epidermidis* (Figure S10, Supporting Information), that lack the carotenoids.

### 2.6 STX Photolysis and Reactive Oxygen Species (ROS) Attack Synergistically Eliminate Intracellular MRSA

Studies dating back to the 1970s have demonstrated that MRSA is able to invade and survive inside mammalian cells, particularly within macrophages.^5^ Though macrophages secrete small effector molecules, including ROS, bacteria including MRSA are capable of neutralizing these effector molecules by producing antioxidants such as STX.^29^ Meanwhile, antibiotics are generally ineffective at clearing intracellular MRSA in large part due to the shield of phagocytic membrane, which poses an alarming threat to the host cells.^5^ As we have demonstrated that STX photolysis plus H_2_O_2_ kill MRSA synergistically, we wondered whether 460 nm light could synergize with the ROS inside macrophage cells to eliminate intracellular MRSA (illustrated in Figure 5f). To evaluate this point, we first infected macrophage cells with MRSA for 1 h. Then, the infected macrophages were exposed to 2 min 460 nm light (48 J cm^−2^) twice over a 6 h interval. Treated macrophages were subsequently lysed to enumerate CFU mL^−1^ of MRSA (spread plates shown in Figure 5f). Figure 5g compiled the statistical analysis of CFU mL^−1^ from different groups. Compared to control group, one‐log_10_ reduction in CFU mL^−1^ was found in the 460 nm light‐treated group. In contrast, high‐concentration vancomycin (5× MIC) was unable to eliminate intracellular MRSA (Figure 5f,g). Additionally, we found that fresh whole blood could eradicate most of MRSA after STX photolysis by blue light (Figure S11, Supporting Information). These findings collectively suggest STX photolysis could assist macrophage cells to eradicate intracellular MRSA.

### 2.7 STX Photolysis and Hydrogen Peroxide Efficiently Eradicate Stationary‐Phase MRSA, Persisters, and *S. aureus* in Biofilms

**Figure 6:**
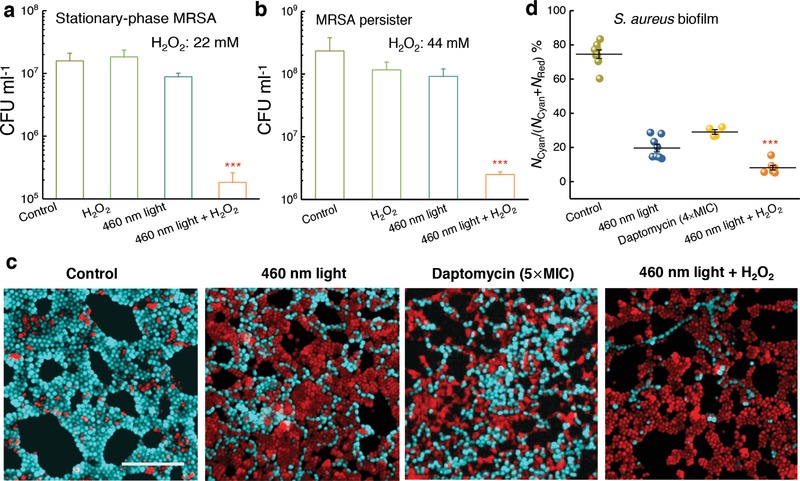
Staphyloxanthin photolysis and H_2_O_2_ effectively eliminate stationary‐phase MRSA, persisters, and *S. aureus* inside a biofilm. a) CFU mL^−1^ of stationary‐phase MRSA after various treatments. Dose: H_2_O_2_, 22 × 10^−3^ m, 460 nm light, 120 J cm^−2^ (*N* = 3). b) CFU mL^−1^ of MRSA persisters after various treatments. Dose: H_2_O_2_, 44 × 10^−3^ m, 460 nm light, 120 J cm^−2^ (*N* = 3). c) Fluorescence images of *S. aureus* with intact (cyan) and damaged cell membrane (red) inside a biofilm after various treatments. Scale bar = 10 μm. 460 nm light: 30 min exposure, 360 J cm^−2^. H_2_O_2_: 0.045%, 20 min culture, then quenched by 0.5 mg mL^−1^ catalase solution. d) Statistical analysis of survival percent of *S. aureus* inside the biofilms. Survival percent =*N*_Cyan_/(*N*_Cyan_ + *N*_Red_), where *N*_Cyan_ and *N*_Red_ represent the number of *S. aureus* with intact and damaged cell membrane, respectively. Error bars show SEM from at least three replicates. Unpaired two‐tailed *t*‐test (***: *p* < 0.001), *** indicates significant difference from the other three groups.

Besides residing inside host immune cells, *S. aureus* is capable of entering the stationary phase or becoming multidrug‐tolerant persisters^30^ to undermine the effectiveness of antibiotics. Persisters could escape the effects of antibiotics without having genetic change.^31^ Moreover, persisters appear to be a major cause of chronic infections since those cells remain less sensitive to antibiotics.^31^ To investigate whether STX photolysis could potentiate low‐concentration H_2_O_2_ to eradicate persister cells, logarithmic‐phase MRSA USA300 were incubated with 10× MIC ciprofloxacin (Figure S12, Supporting Information) for 6 h to kill active cells in order to generate persister cells (Figure S13, Supporting Information).^32^ Stationary‐phase MRSA was obtained by culturing MRSA in medium for 24 h. Then, different treatments subsequently were employed accordingly. It turned out that STX photolysis combined with low‐concentration H_2_O_2_ reduced CFU mL^−1^ by around two orders of magnitude compared to other groups in the case of both stationary‐phase MRSA (**Figure** **6**a) and persisters (Figure 6b). This effectiveness provides clues to treat chronic infections.

*S. aureus* could also form recalcitrant biofilms to evade antibiotics.^33^ Due to the difficulties for antibiotics to penetrate the biofilm matrix termed as extracellular polymeric substances,^33^ bacterial biofilms present a significant source of treatment failure and recurring infections.^33^ Compared to antibiotics, an unparalleled advantage of our photolysis therapy lies in the fact that photons can readily penetrate through a cell membrane or a biofilm, or even a layer of tissue. To explore whether STX photolysis combined with H_2_O_2_ could eradicate *S. aureus* inside a biofilm, we grew biofilms on the bottom of a poly‐lysine‐coated glass dish and then applied 460 nm light or daptomycin (positive control) to treat these biofilms. Then we stained the treated biofilms with live/dead fluorescence probes (Supporting Information), and conducted confocal laser scanning microscopy to examine the efficacy of the above treatments. Figure 6c shows that 460 nm light alone (dose: 360 J cm^−2^) traumatized *S. aureus* by 80%. Figure 6d shows that 460 nm light (dose: 360 J cm^−2^) plus H_2_O_2_ (0.045%, 20 min culture time) reduced *S. aureus* CFU mL^−1^ by 92%. Notably, daptomycin (5× MIC, 24 h culture time) only eliminated *S. aureus* CFU mL^−1^ by 70% (Figure 6d). These results imply an effective way to eradicate *S. aureus* biofilms grown on a medical implant or a host tissue.

### 2.8 STX Photolysis and H_2_O_2_ Effectively Reduce MRSA Burden in two MRSA‐Induced Mice Wound Models

The promising results obtained from the above in vitro studies led us to evaluate the efficacy of STX photolysis in an MRSA‐infected animal model. Skin infections such as diabetic foot ulceration and surgical site infections^34^ are common causes of morbidity in healthcare settings. Notably, *S. aureus* accounts for 40% of these infections.^35^ To optimize the parameters for the in vivo experiment, we first proved that 2 min 460 nm light exposure (dose: 24 J cm^−2^) could cause significant reduction in survival percent of MRSA (Figure S14a, Supporting Information). Then, two times higher antimicrobial efficiency was obtained when cultured with H_2_O_2_ (20 min culture time, 0.045%) subsequently. Furthermore, 5 min culture with H_2_O_2_ after 2 min 460 nm light exposure (dose: 24 J cm^−2^) effectively eliminated MRSA by 60% (Figure S14b, Supporting Information). Notably, the 460 nm light dose applied to treat mouse wound infection was well below the ANSI safety limit for skin exposure.^36^ These parameters were used to apply our photolysis treatment to a MRSA‐infected animal model.

**Figure 7:**
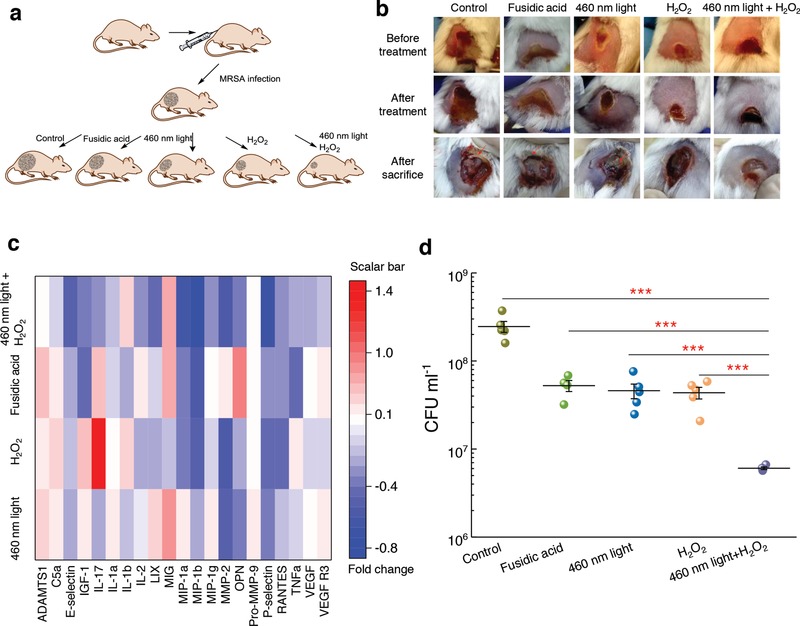
Staphyloxanthin photolysis and H_2_O_2_ effectively reduce MRSA burden in an MRSA‐infected mice wound model. a) Schematic of experiment design (not drawn to scale). b) Pictures of mice wounds of five different groups taken before treatment, after treatment, and after sacrifice. Red arrows indicate pus formation. c) Heat map of key proinflammatory cytokines and markers in the tissue homogenate samples obtained from mice treated with 460 nm light, H_2_O_2_, 460 nm light plus H_2_O_2_, or fusidic acid. Red box indicates upregulation; blue box indicates downregulation; white indicates no significant change. Scale bar represents fold change compared to the untreated group. d) MRSA CFU mL^−1^ after no treatment (control) or three‐consecutive‐day treatment with 2% fusidic acid (petroleum jelly as vehicle), 460 nm light, H_2_O_2_, and 460 nm light plus H_2_O_2_. Dose: 460 nm light, 24 J cm^−2^, H_2_O_2_, 0.045%. Error bars show the SEM from five replicates. Outlier was removed through Dixon's *Q* test. Unpaired two‐tailed *t*‐test (***: *p* < 0.001).

To induce skin lesions in mice (5 groups; 5 mice per group), we severely irritated mice skin by an intradermal injection containing 10^8^ CFU of MRSA USA300 (**Figure** **7**a), the leading source of *S. aureus* induced skin and soft tissue infections in North America.^37^ 60 h postinjection, wound formed at the site of infection (Figure 7b, top). Topical treatments were subsequently administered to each group, twice daily for three consecutive days. Wounds of all the treated groups appeared healthier compared to the control group (Figure 7b, middle). Then, mice were humanely euthanized, and wound tissues were aseptically removed in order to quantify the burden of MRSA in wounds (Supporting Information). We further examined the physiological condition of the wounds. The untreated, fusidic acid‐treated (positive control), and 460 nm light‐treated groups all showed the formation of pus below the wound, in which dead tissues, bacteria, macrophages, and neutrophils dwell.^38^ This symptom suggests that mice immune system fought against the bacteria residing inside the wound tissue. In contrast, mice receiving only H_2_O_2_ or 460 nm light plus H_2_O_2_ treatment exhibited clean wounds that were free of purulent material, swelling, and redness around the edge of the wound (Figure 7b, bottom).

To quantify the anti‐inflammatory effect, we evaluated a panel of cytokines present in the supernatant of homogenized tissues extracted from the wounds of mice. By analyzing the skin homogenate collected from the MRSA mice wound model, we found the highest percent of negative fold change from around 200 kinds of cytokines in the 460 nm light plus H_2_O_2_‐treated group compared to the other groups (Figure S15, Supporting Information). Noteworthily, the 460 nm light plus H_2_O_2_‐treated group demonstrated the highest ratio of decreased expression of these proinflammatory cytokines (Figure 7c). Specifically, a significant decrease was observed in the 460 nm light plus H_2_O_2_‐treated group regarding to key proinflammatory cytokines (TNF‐α, IL‐1α, IL‐2, IL‐17, MIP‐1α, MIP‐1β, LIX) compared with the other groups. Furthermore, there was decreased expression of vascular endothelial growth factor receptor 3 (VEGF R3) in samples obtained from the 460 nm light plus H_2_O_2_‐treated group. This marker is overexpressed in chronic inflammatory wounds, thus resulting in impaired wound reconstruction.^39^ These results support a significantly decreased inflammation in the wounds of mice treated with 460 nm light plus H_2_O_2_, which may suggest that few amount of MRSA exist in the wound tissue.

To quantify the burden of MRSA in wounds, wound tissues were homogenized, and inoculated onto mannitol salt agar plates (*S. aureus* specific) for CFU counting. The results showed that the 460 nm light plus H_2_O_2_‐treated group exhibited significant MRSA reduction compared to all other groups (Figure 7d). Remarkably, the 460 nm light plus H_2_O_2_‐treated group showed more than 1.5‐log_10_ reduction of CFU mL^−1^ compared to the untreated group, and more than one‐log_10_ reduction compared to the fusidic acid‐treated group (Figure 7d). Together, these results demonstrate that the 460 nm light sensitizes MRSA in a skin infection to H_2_O_2_, and provides a more effective treatment than antibiotics.

**Figure 8:**
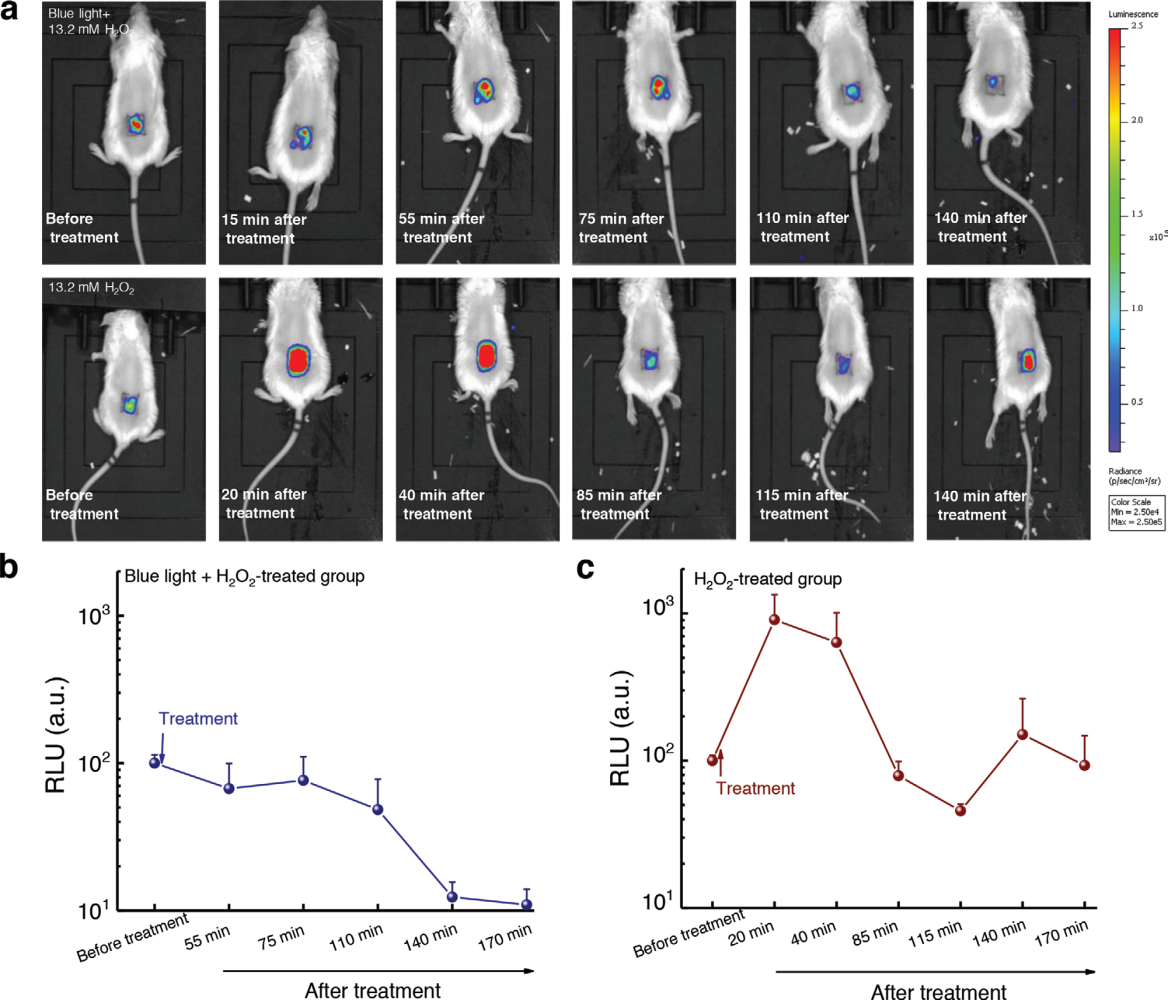
In vivo bioluminescence imaging of MRSA‐infected mice abrasion model under the indicated treatments. a) Representative bioluminescence images of MRSA‐infected mice at different time points after the treatments. Blue light: 460 nm, 120 J cm^−2^ (10 min irradiation). H_2_O_2_: 13.2 × 10^−3^ m. b,c) Quantitative analysis of the bioluminescence signal of MRSA USA300 inside the mice wound after treatment by 460 nm light plus H_2_O_2_ b) and treatment by H_2_O_2_ c), respectively (*N* = 5). Error bars show the SEM.

To confirm the therapeutic effectiveness of our phototherapy, we further utilized a bioluminescent MRSA USA300 strain for in vivo monitoring of MRSA burden in a mouse abrasion model (see Methods in the Supporting Information). The bioluminescence signal from this luminescent MRSA strain is proportional to the number of live bacteria, thus allowing real‐time monitoring of the therapeutic efficacy. After being infected for 3 h, the mice wounds were applied with hydrogen peroxide and 460 nm light plus hydrogen peroxide, respectively. Noteworthily, we found that MRSA burden in the synergy‐treated group has one‐log reduction after treatment (**Figure** **8**a,b). In the group of hydrogen peroxide‐treated group, MRSA burden reduced at the beginning. However, it rapidly recurred back (Figure 8a,c). The synergy between STX photolysis and H_2_O_2_, as shown here, implies a new way to effectively clean patients' wounds or surgical room sterilization.

## 3 Conclusion

Through label‐free transient absorption imaging of chromophores in MRSA, we find that STX, the golden pigment inside *S. aureus*, is prone to photolysis, especially in the blue light region. We further find that STX photolysis transiently elevates membrane permeability for small molecules. Based on these findings, efficient elimination of MRSA is achieved by combining STX photolysis with subsequent ROS attack both in vitro and in vivo. STX photolysis combined with low‐concentration hydrogen peroxide efficiently inactivates slow‐growing stationary‐phase cells and MRSA persisters. Owing to the advantageous light penetration capability compared to antibiotics, STX photolysis could not only assist macrophage cells to eliminate intracellular MRSA, but also reduce the number of sessile bacteria inside biofilms when combined with hydrogen peroxide. Effectiveness of this synergistic treatment is demonstrated in two mice wound infection models. These findings suggest a new way for treating *S. aureus*‐caused infections in clinic, e.g., diabetic ulcerations. Noting that pigmentation is a hallmark of multiple pathogenic microbes,^40^ our work shows the exciting potential of treating multidrug‐resistant bacteria by exploiting the unique photochemistry and photophysics of their intrinsic pigments.

## 4 Experimental Section

Detailed experimental methods and assays are illustrated in the Supporting Information. For the subcutaneous mice wound infection model, this animal experiment was conducted following protocols approved by Purdue Animal Care and Use Committee (PACUC). For the mice abrasion model, this animal experiment was approved by the Subcommittee on Research Animal Care (IACUC) of Massachusetts General Hospital and were in accordance with National Institutes of Health guidelines.

## Conflict of Interest

The authors declare no conflict of interest.

## Supporting information

Supplementary

Supplementary
