# Supplementary material for: Photolysis of Staphyloxanthin in Methicillin‐Resistant Staphylococcus aureus Potentiates Killing by Reactive Oxygen Species
Source: Adv Sci (Weinh). 2019 Mar 30;6(11):1900030. doi: 10.1002/advs.201900030 (PMC6548961; doi:10.1002/advs.201900030)
Supplement: Supplementary file 1 — Supplementary [file ADVS-6-1900030-s002.pdf]

Copyright WILEY-VCH Verlag GmbH & Co. KGaA, 69469 Weinheim, Germany, 2019.

ADVANCED SCIENCE
Open Access

Supporting Information

for *Adv. Sci.,* DOI: 10.1002/advs.201900030

Photolysis of Staphyloxanthin in Methicillin-Resistant *Staphylococcus aureus* Potentiates Killing by Reactive Oxygen Species

Pu-Ting Dong, Haroon Mohammad, Jie Hui, Leon G. Leanse, Junjie Li, Lijia Liang, Tianhong Dai, Mohamed N. Seleem, and Ji-Xin Cheng**

Supplementary Information for

Photolysis of Staphyloxanthin in Methicillin-resistant Staphylococcus aureus Potentiates Killing by Reactive Oxygen Species

Pu-Ting Dong, Haroon Mohammad, Jie Hui, Leon G. Leanse, Junjie Li, Lijia Liang, Tianhong Dai, Mohamed N. Seleem*, and Ji-Xin Cheng*

* To whom correspondence should be addressed: Ji-Xin Cheng (jxcheng@bu.edu) and Mohamed N. Seleem (mseleem@purdue.edu).

This supplementary file includes:

Experimental methods

Figures S1-S15 and captions

Caption for Movie S1

Methods

Transient absorption microscope

An optical parametric oscillator synchronously pumped by a femtosecond pulsed laser generates the pump (1040 nm) and probe (780 nm) pulse trains (Supplementary Fig. 1). The pump (1040 nm) is then frequency-doubled via the second harmonic generation (SHG) process to 520 nm through a barium borate (BBO) crystal. Temporal delay between the pump and probe pulses is controlled through a motorized delay stage. The pump beam intensity is modulated with an acousto-optic modulator (AOM). The intensity of each beams is adjustable through the combination of a half-wave plate (HWP) and a polarization beam splitter (PBS). Thereafter, pump and probe beams are collinearly combined and directed into a lab-built laser-scanning microscope. Through the nonlinear process in the sample, the modulation of pump beam is transferred to the un-modulated probe beam. Computer-controlled scanning galvo mirrors are used to scan the combined laser beams in a raster scanning approach to create microscopic images. The transmitted light is collected by an oil condenser. Subsequently, the pump beam is spectrally filtered by an optical filter and the transmitted probe intensity is detected by a photodiode. A phase-sensitive lock-in amplifier then demodulates the detected signal. Therefore, pump-induced transmission changes in probe beam versus the temporal delay can be measured. This change over time delay shows different time-domain signatures of a chromophore, thus offering the origin of the chemical contrast.

Portable staphyloxanthin photobleaching apparatus

The apparatus is comprised of three parts: a blue light LED (M470L3, Thorlabs), an adjustable collimator (SM1P25-A, Thorlabs), and a power controller (LEDD1B, Thorlabs). The blue light LED has a central emission wavelength of 460 nm with a full width at half maximum of 30 nm. The beam size is adjustable through the collimator (SM1P25-A, Thorlabs). The maximal power of the blue light LED is 200 mW/cm^2.

Carotenoids extraction from MRSA USA300 and acquisition of absorption spectrum

The pigment extraction protocol was adapted from a previous report.[1] Briefly, 100 μL of bacteria solution supplemented with 1900 μL sterile Tryptic Soy Broth (TSB) was cultured for 24 hours with shaking (speed of 250 rpm) at 37 °C. The suspension was subsequently centrifuged for two minutes at 7,000 rpm, washed once, and re-centrifuged. The pigment was extracted with 200 μL methanol at 55 °C for 20 minutes. Extracts from the CrtM mutant were extracted by the same method. The protocol for the treatment of MRSA USA300 with naftifine was adapted from a published report.[2] Bacteria were cultured with 0.2 mM naftifine for 24 hours at 37°C with the shaking speed of 250 rpm. The extraction procedure was the same as described above. The extracted solutions were subsequently exposed to 460 nm light (90 mW, aperture: 1 cm × 1 cm) at different time intervals (0 min, 5 min, 10 min, 20 min). Absorption spectra of the above solutions were obtained by a spectrometer (SpectraMax, M5).

Mass spectrometry

To study the photobleaching chemistry, we extracted crude STX solution from MRSA and exposed the extract to 460 nm light using the procedure described above. The separation was performed on an Agilent Rapid Res 1200 HPLC system. The HPLC-MS/MS system consisted of a quaternary pump with a vacuum degasser, thermostated column compartment, auto-sampler, data acquisition card, and triple quadrupole (QQQ) mass spectrometer (Agilent Technologies, Palo Alto, CA, USA). An Agilent (ZORBAX) SB-C8 column (particle size: 3.5 μm, length: 50 mm, and internal diameter: 4.6 mm) was used at a flow rate of 0.8 mL/min. The mobile phase A was water with 0.1% formic acid and mobile phase B was acetonitrile with 0.1% formic acid. The gradient increased linearly as follows: 5% B, from one to five min; 95% B from five to six min, and 5% B. Column re-equilibration was 6-10 min, 5% B. The relative concentration of STX was quantified using MS/MS utilizing the Agilent 6460 QQQ mass spectrometer with positive electrospray ionization. Quantitation was based on multiple reaction monitoring. Mass spectra were acquired simultaneously using electrospray ionization in the positive modes over the m/z range of 100 to 1000. Nitrogen was used as the drying flow gas.

In order to understand how STX degrades when exposed to 460 nm light, an Agilent 6545 quadrupole time-of-flight (Q-TOF) (Agilent, Santa Clara, CA, USA) was exploited to conduct the separation and quantification steps. This ultra-performance liquid chromatography (UPLC)-MS/MS utilized an Agilent (ZORBAX) SB-C8 column (particle size: 3.5 μm, length: 50 mm, and internal diameter: 4.6 mm) to conduct the separation at a flow rate of 0.8 mL/min. The relative concentration of STX was quantified using MS/MS utilizing the Agilent 6545 Q-TOF MS/MS with positive electrospray ionization. The mobile phase was composed of water (A) and acetonitrile (B). The gradient solution with a flow rate of 0.8 mL/min was performed as follows: 85% B, from 0 to 30 min; 95% B, from 30 to 31 min; 85% B, from 31 to 35 min; 85% B, after 35 min. The sample injection volume was 20 μL. The UPLC-MS/MS analysis was performed in positive ion modes in the m/z range of 100-1100.

In vitro assessment of synergy between 460 nm light and H_2O_2

MRSA USA300 was overnight cultured in sterile tryptic soy broth (TSB) in a 37°C incubator with shaking (at 250 rpm) (OD_{600} = 1). Thereafter, an aliquot (20 μL) of the bacterial suspension was transferred onto a glass slide. Samples were exposed to 460 nm light at different time lengths and variable light intensities. For groups treated with H_2O_2, bacteria were collected in phosphate buffered saline (PBS) supplemented with H_2O_2 at different concentrations (0 mM, 1.4 mM, 2.8 mM, 5.5 mM, 11 mM, 22 mM, 44 mM, 88 mM and 176 mM). The solutions were cultured for 30 min. The solution was serially diluted in sterile PBS and transferred to TSB plates in order to enumerate the viable number of MRSA colony-forming-units (CFU). Plates were incubated at 37 °C for 24

hours before counting viable CFU/mL. Data are presented as viable MRSA CFU/mL and percent survival of MRSA CFU/mL in the treated groups.

Checkerboard broth dilution assays

To evaluate the combinatorial behavior between staphyloxanthin photobleaching and hydrogen peroxide, we performed checkerboard broth dilution assays[3] to calculate a fractional inhibition centration index (FICI). After being exposed to a series of light doses, 0, 15, 30, 60, 120, 240, 480 J/cm^2, MRSA inoculum were transferred to a 96-well plate containing an eight two-fold dilution of hydrogen peroxide starting at 88 mM. Then the plate was cultured at 37°C for 16-20 hours. The optical density at 600 nm (OD_{600}) was recorded to represent the bacteria number. A heat map correlated with OD600 was generated to calculate FICI.

Stationary-phase MRSA and MRSA persister cells

Stationary-phase samples were prepared in the following way: bacteria cells of streaked plate from frozen stock were grown at 37°C, 200 r.p.m. in TSB overnight. Then spin down, wash the pellet with sterile 1×PBS twice. Then the pellet was suspended in 1×PBS.

Persister preparation: As mentioned elsewhere,[4] MRSA USA300 was grown overnight in tryptic soy broth, then diluted in fresh medium and incubated until cells reached logarithmic phase of growth. Bacteria were then exposed to 10×MIC ciprofloxacin for 6 hours. After culturing for 6 hours, spin down and washed the bacteria pellet with sterile 1×PBS twice. Then the washed pellet was resuspended in fresh PBS. Different treatments will be applied by treating the fresh sample with different schemes.

Fluorescence mapping of live and dead S. aureus in biofilm

An overnight culture of S. aureus (ATCC 6538) was grown in a 37 °C incubator with shaking (at 250 rpm). Poly-D-lysine (Sigma Aldrich) was applied to coat the surface of glass bottom dishes (35 mm, In Vitro Scientific) overnight. The overnight culture of S. aureus was diluted (1:100) in TSB containing 5% glucose and transferred to the glass bottom dishes. The plates were incubated at 37°C for 24-48 hours in order to form mature biofilm. Thereafter, the media was removed and the surface of the dish was washed gently with sterile water to remove planktonic bacteria. Plates were subsequently treated with 460 nm light alone (200 mW/cm^2, 30 min), H_2O_2 (13.2 mM, 20 minutes) alone, or a combination of 460 nm light and H_2O_2. Groups receiving H_2O_2 were quenched through addition of 0.5 mg/mL catalase (Sigma Aldrich, 50 mM, pH = 7 in potassium buffered solution). After treatment, biofilms were immediately stained with fluorescence dyes, as follows.

To confirm the existence of biofilm on the glass bottom surface, a biofilm matrix stain (SYPRO® Ruby Biofilm Matrix Stain, Invitrogen) was utilized. Biofilms were stained with the live/dead biofilm viability kit (Invitrogen) for 30 minutes to quantify the survival

percent of S. aureus in the biofilm after treatment. The biofilms were washed with sterile water twice and then imaged using a fluorescence microscope (OLYMPUS BX51, objective: 60×, oil immersion, NA = 1.5). Two different excitation channels (live: FITC; dead: Texas Red) were utilized in order to map the ratio of live versus dead cells within the biofilm. The acquired images were analyzed by ImageJ (National Institute of Health). Statistical analysis was conducted via a two-paired t-test through GraphPad Prism 6.0 (GraphPad Software, La Jolla, CA).

Confocal laser scanning microscopy to image intracellular hydrogen peroxide

S. aureus (ATCC 6538) was utilized as the experimental strain. An intracellular hydrogen peroxide kit (Sigma Aldrich) was employed to image the intracellular hydrogen peroxide. The excitation of this dye was through an Olympus FV1000 scanning confocal microscope with the excitation line of 488 nm. A 60 × water immersion objective (Olympus) was used. For H_2O_2-treated group, overnight cultured S. aureus was treated with 0.15% H_2O_2 for 20 min. For 460 nm light plus H_2O_2-treated group, overnight cultured S. aureus was treated with 0.15% H_2O_2 for 20 min after 30-min 460 nm light exposure. Then spin down, washed with sterile PBS, repeat twice. Stain the above menioned samples with the intracellular hydrogen peroxide kit for 30 min. Spin down, washed with sterile PBS, repeat twice of this process. Sandwiched washed samples in between two clean coverslides (VWR). Images were processed by ImageJ (National Institute of Health).

Intracellular MRSA infection model

Murine macrophage cells (J774) were cultured in DuTSBecco's Modified Eagle Medium (DMEM) supplemented with 10% fetal bovine serum (FBS) at 37 °C with CO_2 (5%). Cells were exposed to MRSA USA400 at a multiplicity of infection of approximately 100:1. 1-hour post-infection, J774 cells were washed with gentamicin (50 µg/mL, for one hour) to kill extracellular MRSA. Vancomycin, at a concentration equal to 2 µg/mL (4 × MIC, MIC: minimal inhibition concentration), was added to six wells. Six wells received 460 nm light treatment twice (six hours between treatments) for two minutes prior to addition of DMEM + 10% FBS. Three wells were left untreated (medium + FBS) and three wells received dimethyl sulfoxide at a volume equal to vancomycin-treated wells. Twelve hours after the second 460 nm light treatment, the test agents were removed; J774 cells were washed with gentamicin (50 µg/mL) and subsequently lysed using 0.1% Triton-X 100. The solution was serially diluted in PBS and transferred to tryptic soy agar plates in order to enumerate the MRSA CFU present inside infected J774 cells. Plates were incubated at 37 ºC for 22 hours before counting viable CFU/mL. Data are presented as log_{10}(MRSA CFU/mL) in infected J774 cells in relation to the untreated control. The data was analyzed via a two-paired t-test, utilizing GraphPad Prism 6.0 (GraphPad Software, La Jolla, CA).

In vivo MRSA mice wound model

This animal experiment was conducted following protocols approved by Purdue Animal Care and Use Committee (PACUC). To initiate the formation of a skin wound, five groups (N=5) of eight-week-old female BALB/c mice (obtained from Harlan Laboratories, Indianapolis, IN, USA) were disinfected with ethanol (70%) and shaved on the middle of the back (approximately a one-inch by one-inch square region around the injection site) one day prior to infection as described from a reported procedure[5]. To prepare the bacterial inoculum, an aliquot of overnight culture of MRSA USA300 was transferred to fresh tryptic soy broth and shaken at 37 °C until an OD_{600} value of ~1.0 was achieved. The cells were centrifuged, washed once with PBS, re-centrifuged, and then re-suspended in PBS. Mice subsequently received an intradermal injection (40 μL) containing 2.40×10^9 CFU/mL MRSA USA300. An open wound formed at the site of injection for each mouse, ~60 hours post-infection.

Topical treatment was initiated subsequently with each group of mice receiving the following: fusidic acid (2%, using petroleum jelly as the vehicle), 13.2 mM H_2O_2 (0.045%, two-minute exposure), 460 nm light (two-minute exposure, 24 J/cm^2), or a combination of 460 nm light (two-minute exposure, 24 J/cm^2) and 13.2 mM H_2O_2 (two-minute exposure). One group of mice was left untreated (negative control). Each group of mice receiving a particular treatment regimen was housed separately in a ventilated cage with appropriate bedding, food, and water. Mice were checked twice daily during infection and treatment to ensure no adverse reactions were observed. Mice were treated twice daily (once every 12 hours) for three days, before they were humanely euthanized via CO_2 asphyxiation 12 hours after the last dose was administered. The region around the skin wound was lightly swabbed with ethanol (70%) and excised. The tissue was subsequently homogenized in PBS. The homogenized tissue was then serially diluted in PBS before plating onto mannitol salt agar plates. Plates were incubated for at least 19 hours at 37 °C before viable MRSA CFU/mL were counted for each group. Outlier was removed based upon the Dixon Q Test. Data were analyzed via a two-paired t-test, utilizing GraphPad Prism 6.0 (GraphPad Software, La Jolla, CA).

In vivo bioluminescence mice abrasion model

This animal experiment was approved by the Subcommittee on Research Animal Care (IACUC) of Massachusetts General Hospital and were in accordance with National Institutes of Health guidelines. As reported elsewhere,[6] adult female BALB/c mice (Charles River Laboratories, Wilmington, MA, N=5), 7-8 weeks old and weighing 16-18 g. The mice were maintained on a 12 h light/dark cycle at a room temperature of 21°C. Before the creation of skin abrasions, mice were anesthetized by i.p. injection of a ketamine-xylazine cocktail and then shaved on the dorsal surfaces using an electric fur clipper. Mouse skin was then scraped using no. 15 scalpel blades until a redden area appeared. This procedure resulted in first degree skin abrasions with most part of the epidermis removed. One drop (100 μL) of overnight cultured bioluminescent MRSA USA300 LAC::Lux suspension containing around 10^7 CFU were evenly smeared onto

the mice wounds by using a micropipette. Three hours later, different treatment schemes were applied and the real-time bioluminescence signal from each mouse was acquired.

Statistical analysis

Statistical analysis was conducted through unpaired t-test. *** means significantly different with the p-value < 0.001. ** means significantly different with the p-value < 0.01. * means significantly different with the p-value < 0.05. 'ns' means no significance.

Whole blood

After photobleaching of MRSA by 460 nm light, MRSA were either cultured in sterile PBS (control) or whole blood (Innovative Research Inc., Novi, MI) for 9 hours. The efficacy was evaluated through enumerating CFU.

Photobleaching model

To analyze the time-lapse transient absorption signals, we utilized a mathematical model which was originally used to depict the photobleaching of photosensitizers happening during a photodynamic process[7]:

$$\frac{d[C]}{dt} = -k_1[C][R] \qquad (1)$$

, where t is the duration time, [C] is the concentration of chromophore (e.g., carotenoids in S. aureus), $k_1 (k_1 = {}^{1}/_{\tau_1})$ is the rate constant of first-order photobleaching with τ_1 being the first-order photobleaching time constant, [R] is the concentration of active agents (the chromophores which have interaction with light):

$$[R] \sim [R]_0 + k_2[C] \qquad (2)$$

, where k_2 $(k_2 = {}^{1}/_{([C]_0 * \tau_2)})$ is the rate constant of second-order photobleaching with τ_2 being the second-order photobleaching time constant, $[R]_0$ is the initial concentration of the active agent, respectively. The combination of equation (1) and equation (2) leads to:

$$\frac{d[C]}{dt} = -\frac{1}{\tau_1} * [C] - \frac{1}{\tau_2 * [C]_0} * [C]^2 \qquad (3)$$

The solution for equation (3) is:

$$\frac{[C]_t}{[C]_0} = A * \frac{\exp(-\frac{t}{\tau_1})}{1 + \frac{\tau_1}{\tau_2} * (1 - \exp(-\frac{t}{\tau_1})} \qquad (4)$$

, where A is a constant. When first-order photobleaching process pivots (usually happening for low concentration of chromophore and having the involvement of oxygen), $\tau_2 \to \infty$, then equation (4) becomes:

$$\frac{[C]_t}{[C]_0} = A * \exp\left(-\frac{t}{\tau_1}\right) \qquad (5)$$

, which is similar to first-order kinetic reaction. At this occasion, the photobleaching rate is linearly proportional to the concentration of chromophore. When second-order photobleaching process dominates (usually happening for high concentration of chromophore potentially through triplet-triplet annihilation), $\tau_1 \rightarrow \infty$, then equation (4) becomes:

$$\frac{[C]_t}{[C]_0} = A * \frac{1}{1 + \frac{t}{\tau_2}} \qquad (6)$$

Under this condition, the photobleaching rate is proportional to the square of chromophore concentration. According to the fitting result, the decaying signal of MRSA under our transient absorption microscopy settings belongs to a second-order photobleaching with $\tau_1 \rightarrow \infty$.

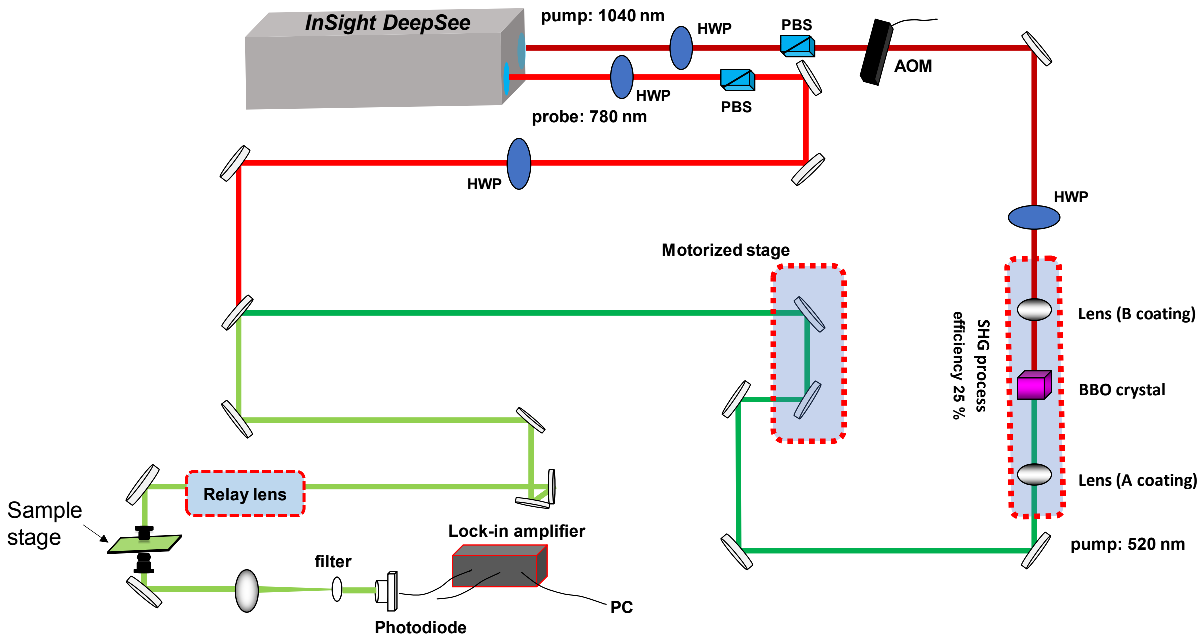

Figure S1. Schematic illustration of transient absorption microscope. HWP, half wave plate. PBS, polarization beam splitter. AOM, acousto-optic modulator. BBO, barium borate crystal. SHG, second harmonic generation. B coating: 600-1050 nm; A coating: 350-700 nm.

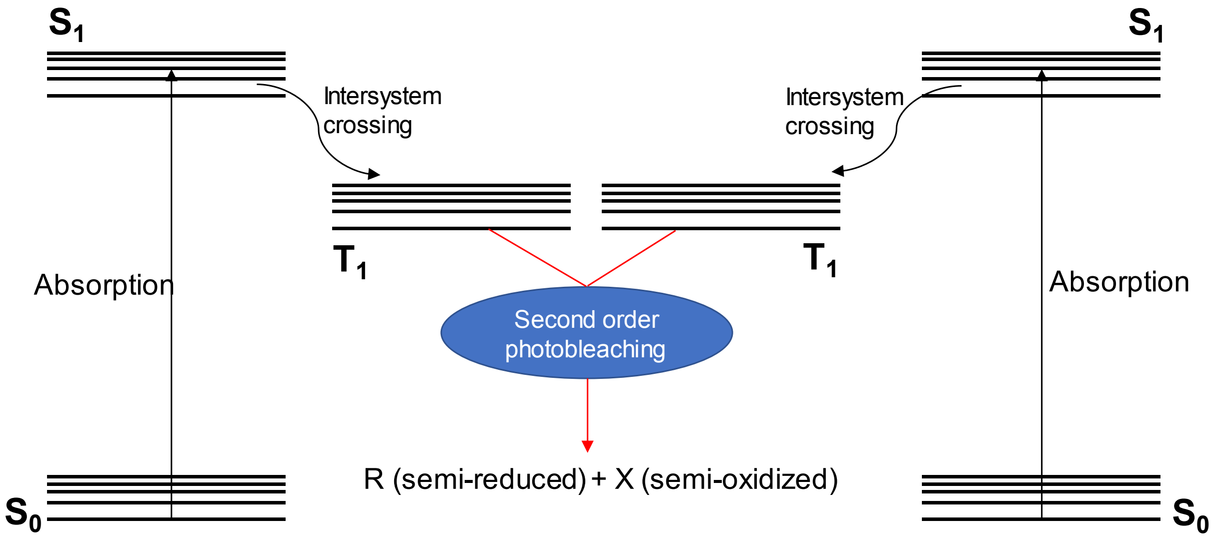

Figure S2. Illustration of second-order photobleaching in Jablonski Diagram. S_0, ground state. S_1, singlet excited state. T_1, triplet excited state.

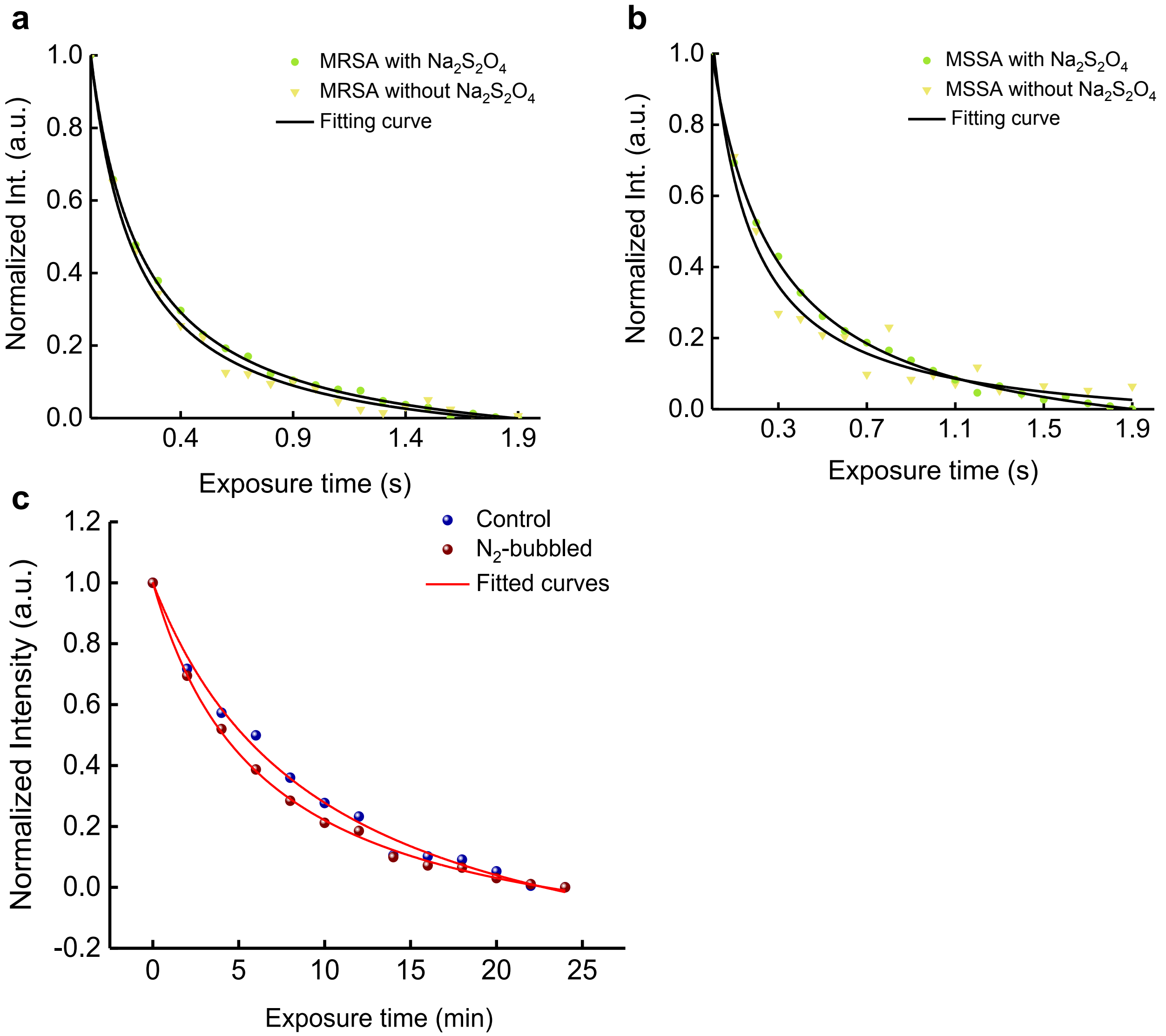

Figure S3. Photobleaching of staphyloxanthin in MRSA under oxygen depletion condition through transient absorption microscopy. (**a**) Time-lapse signals from MRSA with (green dots) or without $Na_2S_2O_4$ (yellow dots). (**b**) Time-lapse signals from methicillin-susceptible S. aureus (MSSA) with (green dots) or without $Na_2S_2O_4$ (yellow dots). $Na_2S_2O_4$: oxygen scavenger, 20 mM. (**c**) Absorption of extracted chromophore solution from MRSA at 460 nm versus blue light (460 nm) exposure time under untreated condition (blue dots) along with 2-hour N_2-bubbled condition (brown dots). Blue light: 460 nm, 160 mW/cm^2. Curves were fitted by equation (1).

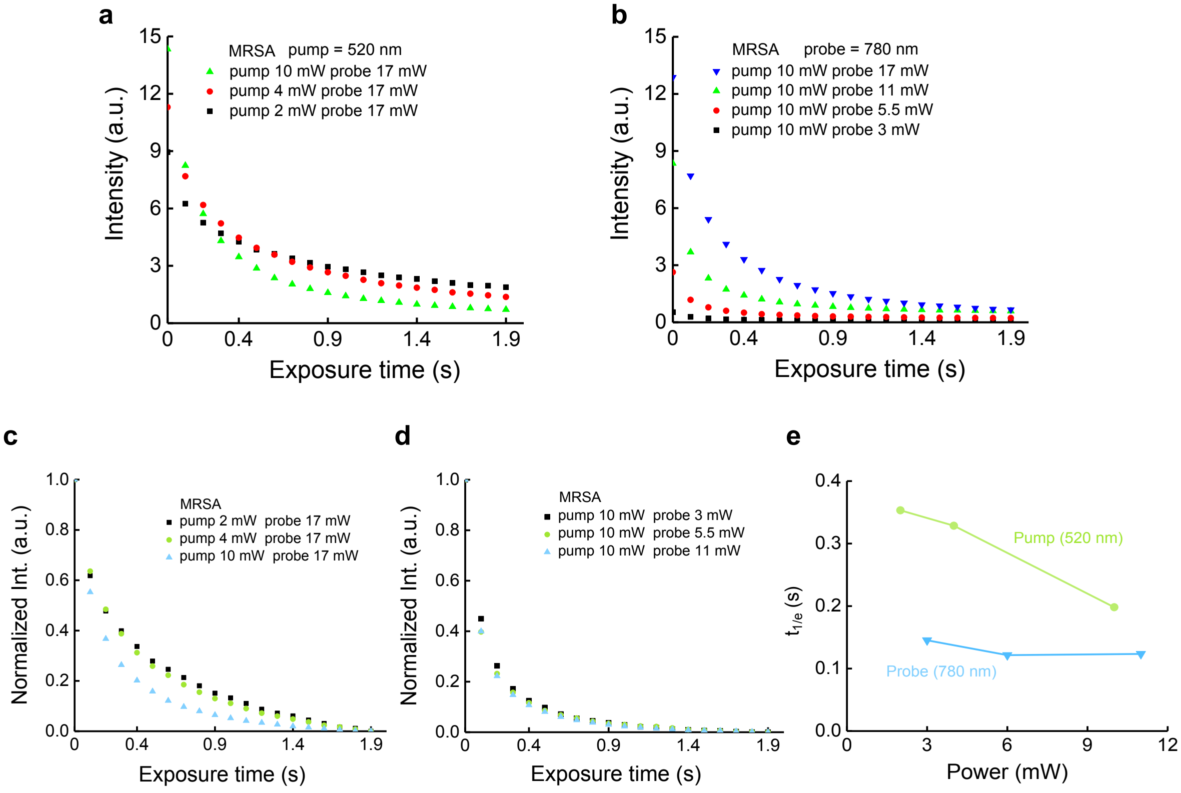

Figure S4. Dependence of time-lapse signal intensity and photobleaching speed on laser wavelength and intensity under transient absorption microscope. Time-lapse signals from MRSA colony with increasing pump power (**a**), and probe power (**b**). Normalized time-lapse signals from MRSA colony with increasing pump power (**c**), and probe power (**d**). (**e**) Pump and probe power dependence of the decay time (at 1/e of its initial intensity (t = 0 s)) from MRSA colony. Pump: 520 nm, probe: 780 nm.

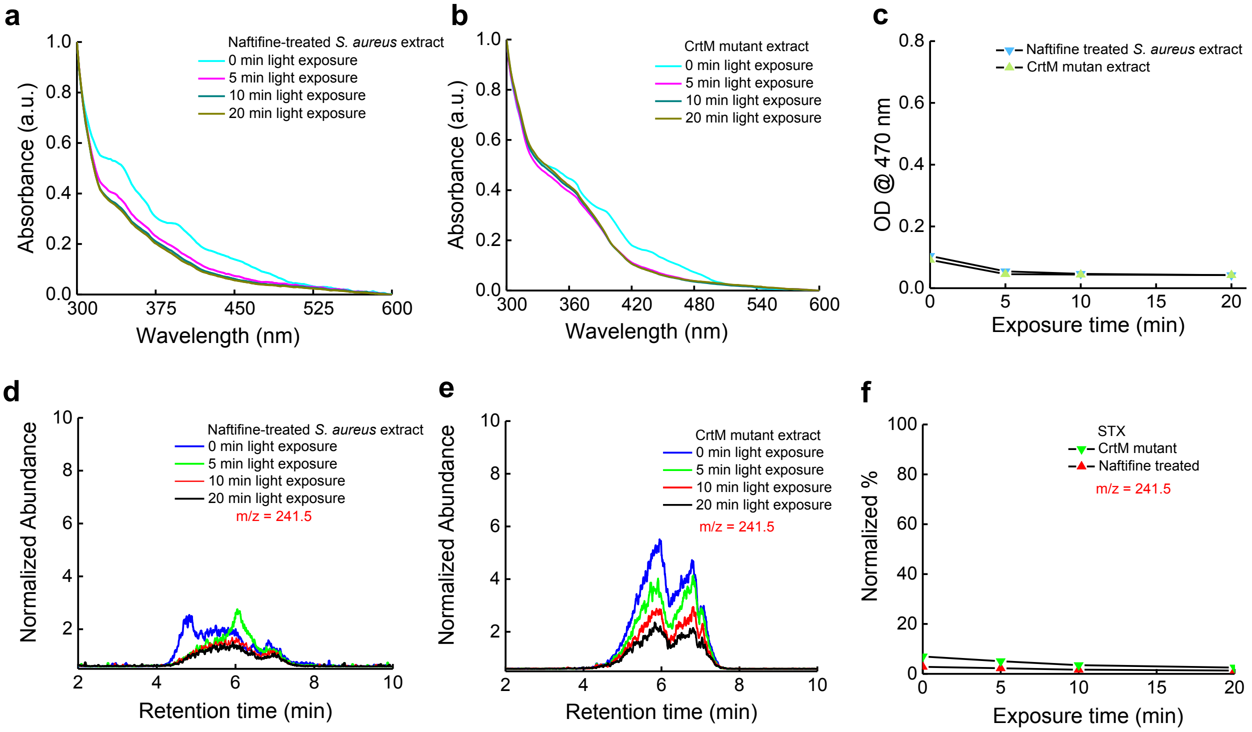

Figure S5. Absorption spectroscopy and mass spectrometry analysis of extracts from naftifine-treated S. aureus and CrtM mutant at different 460 nm light exposure time. Absorption spectrum of naftifine-treated S. aureus (**a**), and CrtM mutant (**b**) extracts at different 460 nm light exposure time. (**c**) OD_{470} (optical density at 470 nm) of extracts from naftifine-treated S. aureus and CrtM mutant at different 460 nm light exposure time. HPLC chromatograms of STX from naftifine-treated S. aureus (**d**) and CrtM mutant (**e**) over 460 nm light exposure time. (**f**) Calculated HPLC area of STX from naftifine-treated S. aureus and CrtM mutant over 460 nm light exposure time, normalized by the amount of STX extracted from untreated S. aureus.

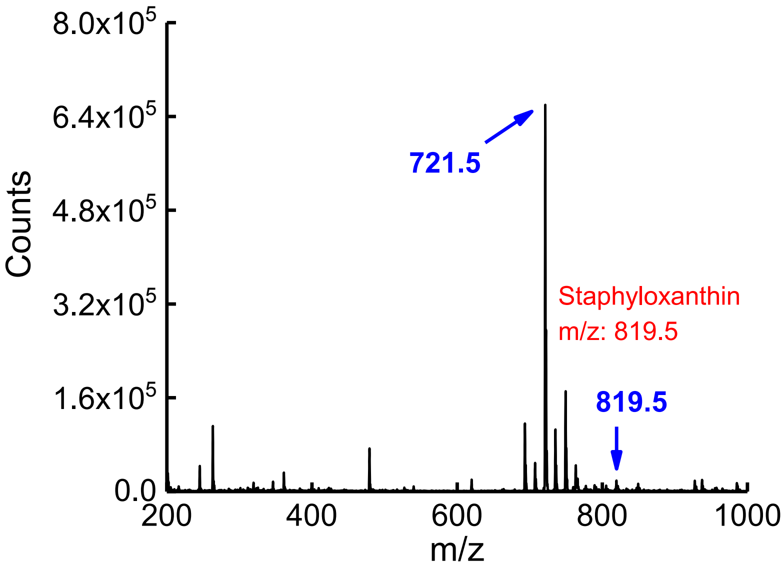

Figure S6. Mass spectrum of S. aureus extract with m/z ranging from 200 to 1000 at a collision energy of 10 eV. The m/z = 819.5 peak corresponds to staphyloxanthin (M_w = 818.5 g/mol).

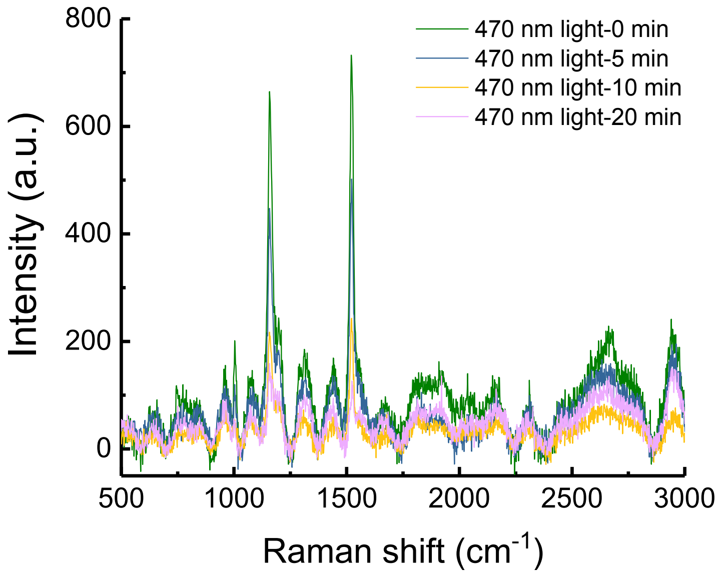

Figure S7. Raman spectrum of MRSA solution under different 460 nm light exposure time. 460 nm light intensity: 200 mW/cm^2. Raman excitation wavelength: 532 nm. Acquisition time: 30 s. Green line: untreated MRSA, blue line: 5-min 460 nm light exposure, yellow line: 10-min 460 nm light exposure, purple: 30-min 460 nm light exposure.

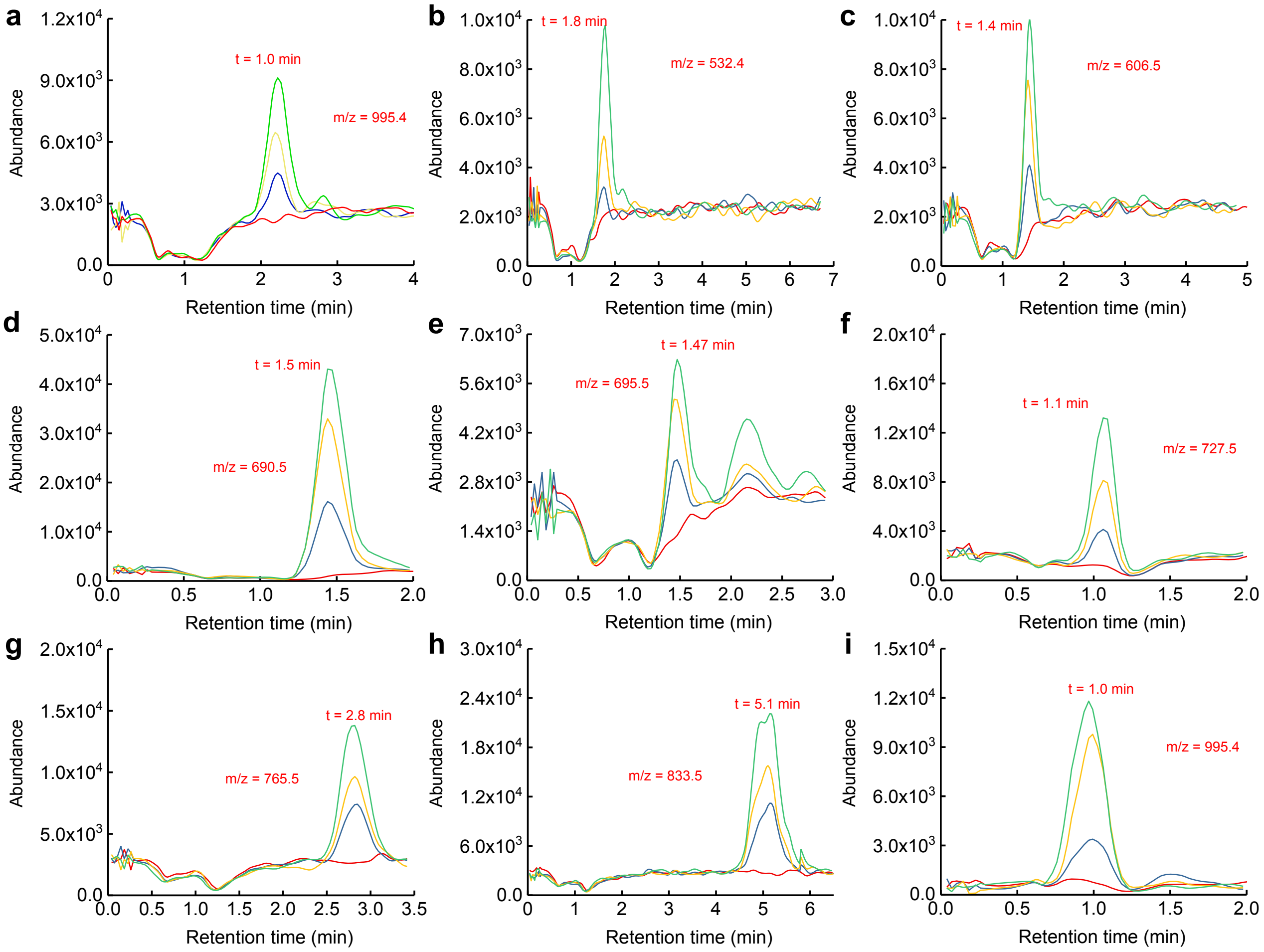

Figure S8. Ultra-performance liquid chromatography (UPLC) chromatograms of other products from staphyloxanthin photolysis unveiled by time-of-flight MS/MS. Green line corresponds to 10-min 460 nm light exposure; yellow line corresponds to 5-min 460 nm light exposure; blue line corresponds to 2.5-min 460 nm light exposure; red line corresponds to 0-min 460 nm light exposure. 460 nm light: 90 mW/cm^2.

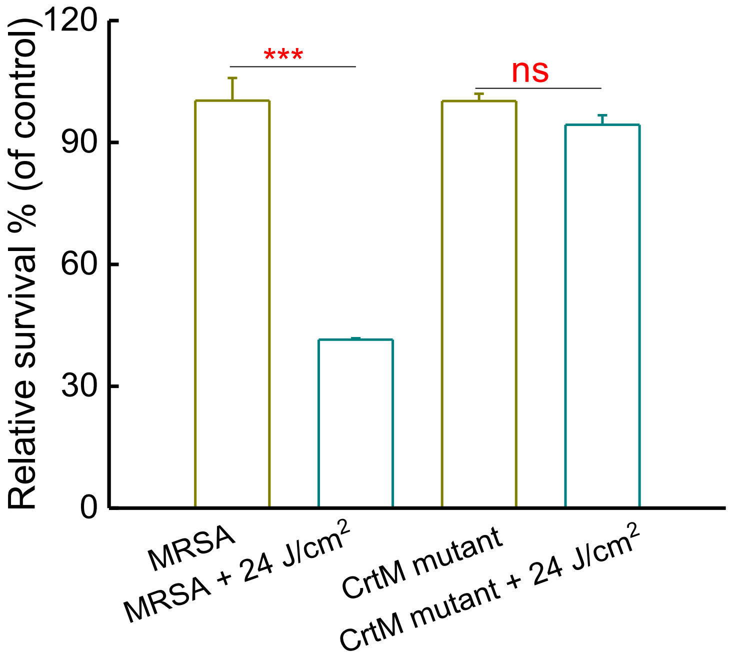

Figure S9. Relative survival percent of MRSA and CrtM mutant S. aureus with or without 460 nm light exposure. Error bar represents standard error of the mean from three replicates. Statistical significance was determined by unpaired two-tailed t-test (***: $p < 0.001$, ns: not significant).

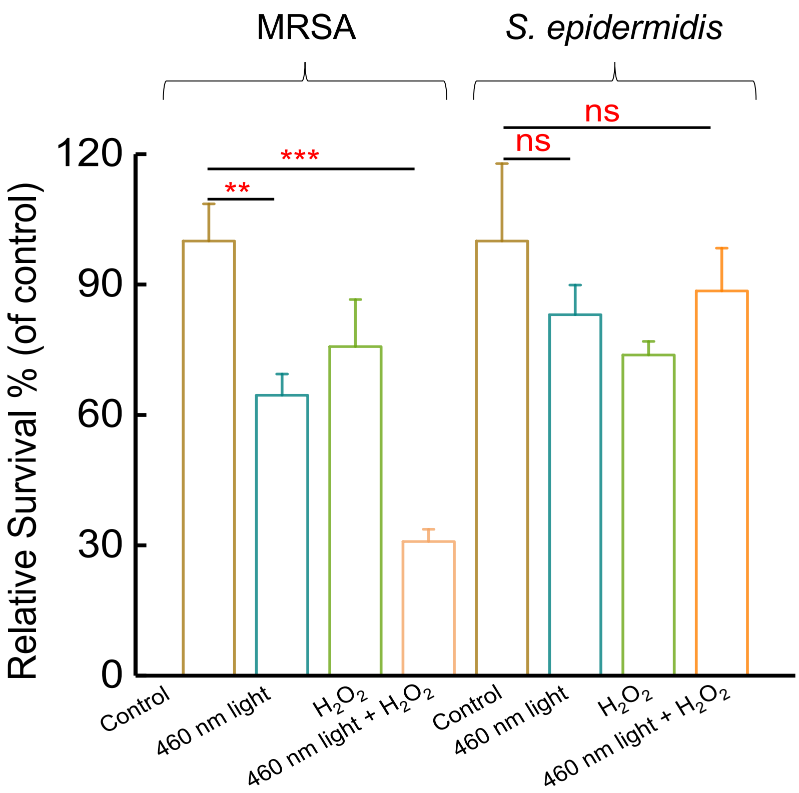

Figure S10. Relative survival percent of MRSA and S. epidermidis under no treatment, treatment of 460 nm light, treatment of H_2O_2, and treatment of 460 nm light plus H_2O_2. Error bar represents standard error of the mean from three replicates. Statistical significance was determined by unpaired two-tailed t-test (***: $p < 0.001$; **: $p < 0.01$, ns means not significant).

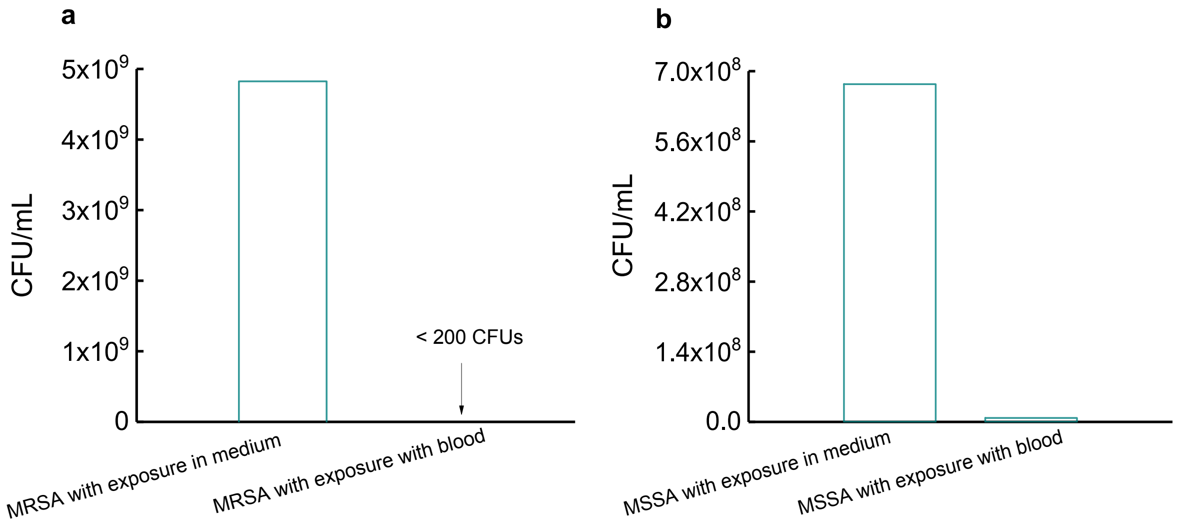

Figure S11. Blue light irradiation assists human whole blood to eradicate MRSA and MSSA. (**a**) CFU results of MRSA cultured in medium and human whole blood after blue light exposure. (**b**) CFU results of MSSA cultured in medium and human whole blood after blue light exposure. Blue light: 440 nm, 10 mW, 1-hour exposure time. In control groups, MRSA and MSSA were cultured in sterile PBS for 9 h. In experimental groups, MRSA and MSSA were cultured in fresh whole blood for 9 h.

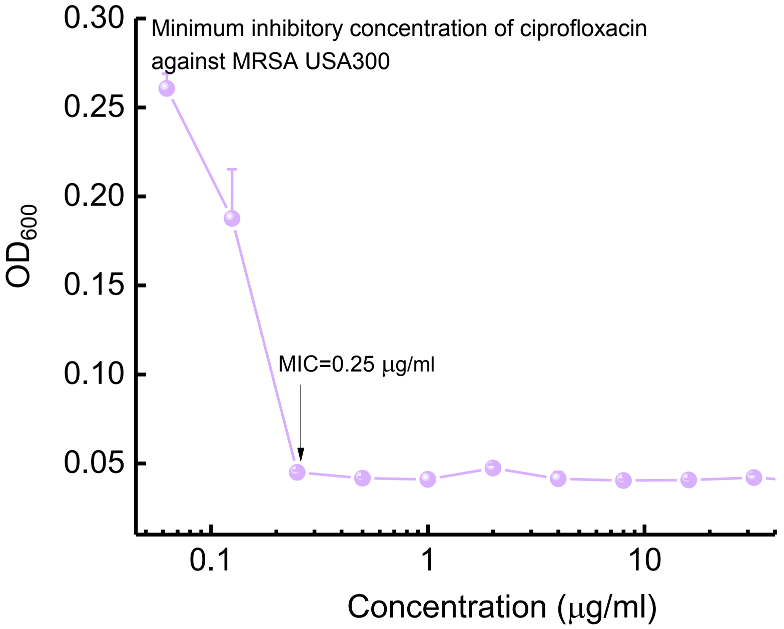

Figure S12. Minimum inhibition concentration of ciprofloxacin (MIC) against MRSA USA300.

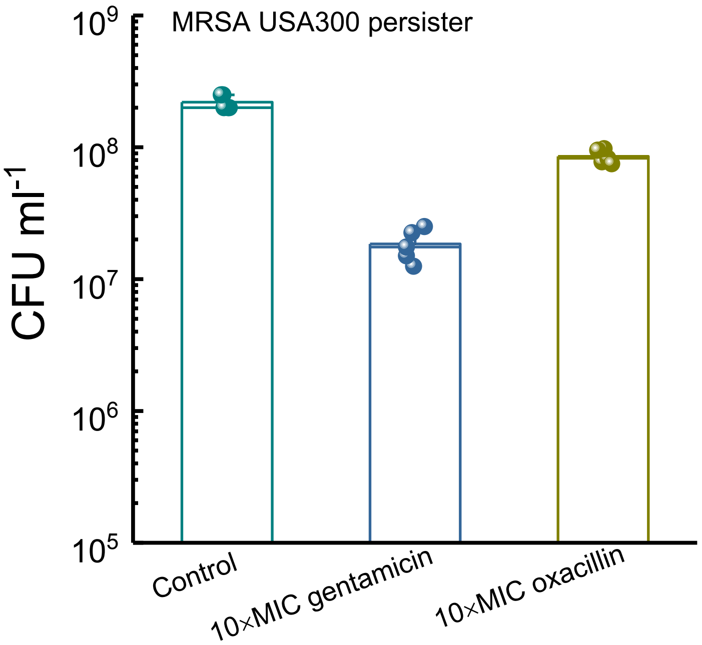

Figure S13. CFU ml^{-1} of MRSA USA300 persister after 6-h treatment with 10 × MIC of gentamicin or oxacillin.

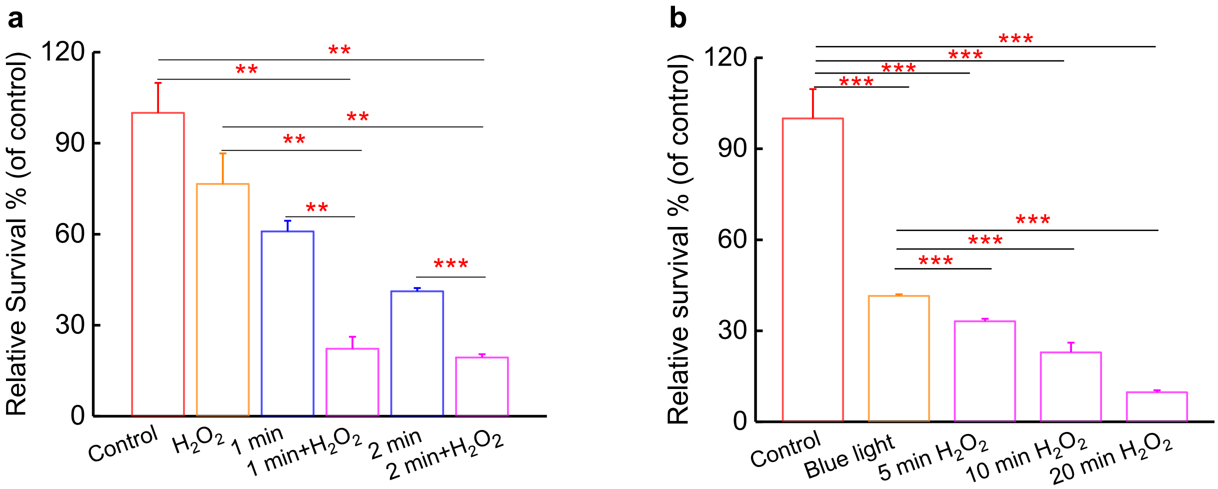

Figure S14. Relative survival percent of MRSA over 460 nm light exposure time and H_2O_2 culture time. 460 nm light: 1 to 2 min (12 to 24 J/cm^2). H_2O_2: 0.045%. (**a**) Results of survival percent of MRSA with fixed H_2O_2 culture time (20-min culture time at 37°C). (**b**) Results of survival percent of MRSA with fixed 460 nm light exposure time (24 J/cm^2). Error bars show standard error of the mean from at least three replicates. Statistical significance was determined by two-paired t-test (***: $p < 0.001$, **: $p < 0.01$, ns: not significant).

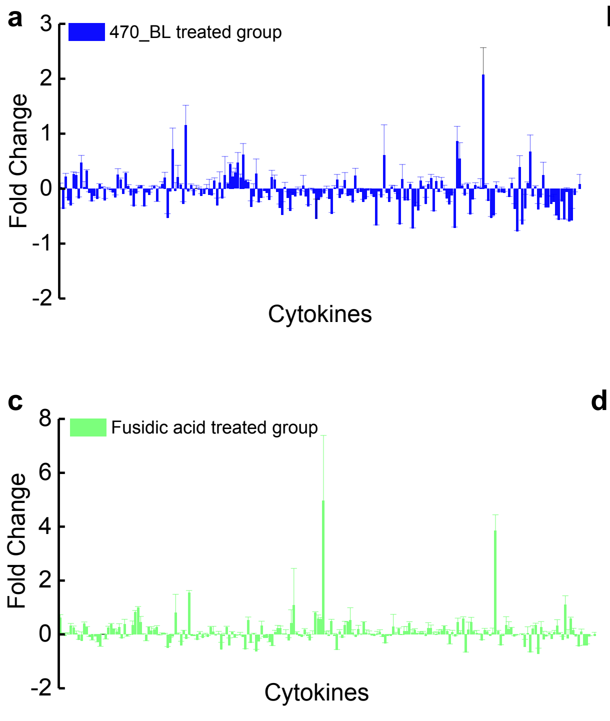

Figure S15. Fold changes from around 200 kinds of cytokines among 460 nm light-treated group (blue), H_2O_2-treated group (yellow), fusidic acid-treated group (green) along with 460 nm light plus H_2O_2-treated group (cyan). N=4.

Movie S1. Real-time visualization of staphyloxanthin photobleaching in MRSA captured by transient absorption microscopy.

References:

[1] G. Y. Liu, A. Essex, J. T. Buchanan, V. Datta, H. M. Hoffman, J. F. Bastian, J. Fierer, V. Nizet, *The Journal of experimental medicine* **2005**, *202*, 209-215.

[2] F. F. Chen, H. X. Di, Y. X. Wang, Q. Cao, B. Xu, X. Zhang, N. Yang, G. J. Liu, C. G. Yang, Y. Xu, H. L. Jiang, F. L. Lian, N. X. Zhang, J. Li, L. F. Lan, *Nat Chem Biol* **2016**, *12*, 174-179.

[3] M. M. Sopirala, J. E. Mangino, W. A. Gebreyes, B. Biller, T. Bannerman, J.-M. Balada-Llasat, P. Pancholi, *Antimicrobial Agents and Chemotherapy* **2010**, *54*, 4678-4683.

[4] M. F. Mohamed, A. Abdelkhalek, M. N. Seleem, *Scientific Reports* **2016**, *6*, 29707.

[5] H. Mohammad, M. Cushman, M. N. Seleem, *PLoS ONE* **2015**, *10*, e0142321.

[6] T. Dai, A. Gupta, Y.-Y. Huang, M. E. Sherwood, C. K. Murray, M. S. Vrahas, T. Kielian, M. R. Hamblin, *Photomedicine and laser surgery* **2013**, *31*, 531-538.

[7] A. A. Stratonnikov, G. A. Meerovich, V. B. Loschenov, in *BiOS 2000 The International Symposium on Biomedical Optics*, International Society for Optics and Photonics, **2000**, pp. 81-91.
